# Rapid traceability of *Gastrodia elata* Blume origins and analysis of key volatile organic components using FTIR and HS-SPME-GC–MS combined with chemometrics

**DOI:** 10.1016/j.fochx.2025.102770

**Published:** 2025-07-09

**Authors:** Yingfeng Zhong, Jieqing Li, Honggao Liu, Yuanzhong Wang

**Affiliations:** a College of Agronomy and Biotechnology, Yunnan Agricultural University, Kunming 650201, China; b Medicinal Plants Research Institute, Yunnan Academy of Agricultural Sciences, Kunming 650200, China; c Yunnan Key Laboratory of Gastrodia and Fungi Symbiotic Biology, Zhaotong University, Zhaotong 657000, Yunnan, China

**Keywords:** *Gastrodia elata* Blume, HS-SPME-GC–MS, VOCs, FTIR, Chemometrics

## Abstract

*Gastrodia elata* Blume (*G. elata*) is highly favored in the edible sector owing to its rich nutritional content and distinct flavor. Herein, headspace solid phase microextraction gas chromatography–mass spectrometry (HS-SPME-GC–MS) and Fourier transform infrared spectroscopy (FTIR) technology were employed to classify the origin of *G. elata* and quantify volatile organic compounds (VOCs). GC–MS revealed that sweet, fruity, and nutty are the key flavor characteristics of *G. elata*, with samples from Zhaotong City, Yunnan Province, exhibiting superior flavor and richness Based on FTIR data, the gray wolf optimizer-support vector machine and residual convolutional neural network achieved 100 % accuracy in *G. elata* traceability, with an F_1_ of 1.000. Additionally, the partial least squares regression model successfully quantified the main components 2-Nonenal and 2(3H)-Furanone, dihydro-5-propyl- in *G. elata*, with prediction set residual deviations of 2.6003 and 2.3883, respectively. This approach offers a novel framework for monitoring VOCs quality control in other foods.

## Highlights

• Propose a new method for tracing the origin of *Gastrodia elata* Bulme.
• Samples from Yunnan Zhaotong were superior in richness and flavor.
• The origin traceability of *Gastrodia elata* Bulme was accurately distinguished.
• The major flavor contributions are 2-Nonenal and 2(3H)-Furanone, dihydro-5-propyl.
• Rapid quantitative models can predict key flavor components.

## Graphical abstract

**Figure:**
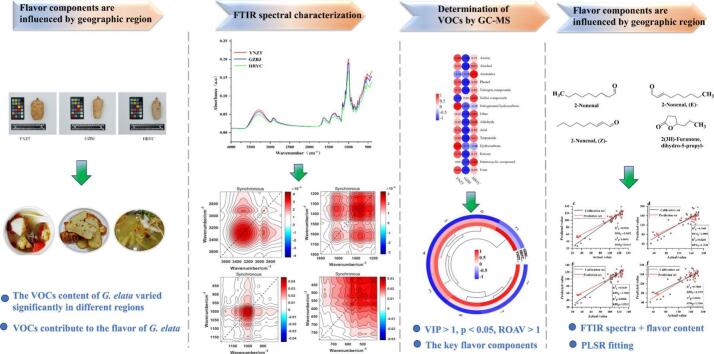

## 1 Introduction

*Gastrodia elata* Blume (*G. elata*), a perennial herb in the Orchidaceae family, is widely distributed across Asian regions, including China, Korea and Nepal (Li et al., 2025). In China, it is primarily distributed in the provinces of Yunnan, Hubei and Guizhou, where it grows in humus-rich, shaded and moist understory environments (Du, Liu, Mei, & Zhan, 2025). The rhizomes of *G. elata* are widely applied in the treatment of hypertension, cerebral injuries, anti-aging, anti-cancer and so on (Gong et al., 2024; Zhong et al., 2025). For example, PGE-40 (1–6 mg/mL) in *G. elata* inhibits MCF-7 cell proliferation, and parishin A and N_6_-(4-hydroxybenzyl) adenine riboside (20 μM) reduce senescence-associated β-galactosidase activity, thereby demonstrating anti-aging effects (Gong et al., 2024). In addition to its medicinal uses, *G. elata* is also valued as a culinary ingredient stewed with meat, fruits and vegetables in some regions of China (e.g., Guizhou and Yunnan Provinces), and is considered a nutritious and flavorful tonic (Wang et al., 2025) (Fig. S1). It has been officially classified as a plant that “traditionally serves as both food and medicinal material” by the State Administration for Market Regulation (SAMR) and the National Health Commission of China (NHC) (Duan et al., 2023; Zhang et al., 2024).

*G. elata* contains abundant polysaccharides, volatile organic compounds (VOCs) and organic acids, which contribute to its special aroma (Wang et al., 2025; Zhong et al., 2025). Its quality and aroma are predominantly determined by the compositional diversity and type of VOCs (Wang et al., 2025). However, geographic origins, variety, harvest season, and processing methods of different *G. elata* can significantly influence the variability of VOCs (Du et al., 2025). Wang et al. (2025) reported clear regional differences in the flavor profiles of *G. elata*, identifying 19 key VOCs. Another study conducted by Duan et al. (2023) highlighted significant inter-regional differences in *G. elata* VOCs across different origins (Yunnan, Sichuan, Shaanxi, Anhui, Hubei and Guizhou). With the advance of the functional food market, *G. elata* deep-processed products have formed a large-scale industry. Notably, the flavor compounds in *G. elata* play dual roles as both core determinants of sensory quality and bioactive carriers that directly affect the functional efficacy of products. This dual property makes *G. elata* flavor research increasingly critical. *G. elata* originating from Zhaotong, Yunnan Province (YNZT) is currently recognized as a premium variety, featured by its superior medicinal efficacy and culinary applications (Zheng, Li, Liu and Wang, 2023; Li et al., 2025b). Due to its high market recognition and consumer preference, YNZT *G. elata* has been granted national geographical indication protection (Zheng et al., 2023). However, given its prominent role in medicine and food, a large number of substandard and counterfeit products have recently flooded the market. Due to notable variations in aroma and quality among *G. elata* from different regions, VOCs-based qualitative and quantitative analysis could offer a novel approach for the assessment of *G. elata*'s quality.

Currently, the conventional assays for food flavor components mainly focus on subjective visual and gustatory evaluations (Duan et al., 2023). With the progress of science, advanced instrumental analytical techniques, such as gas chro-matography-mass spectrometry (GC–MS) and headspace gas chromatography–ion mobility spectrometry (HS-GC-IMS) have been extensively applied for the detection of VOCs in food, beverages and herbs (Ma et al., 2024; Wang et al., 2025; Yuan et al., 2022). Although these techniques offer high reproducibility and accuracy, they are limited by drawbacks such as labor-intensive sample preparation, high reagent consumption, and prolonged analysis times (Deng et al., 2025b; Yu et al., 2024). Attenuated total reflection Fourier transform infrared spectroscopy (ART-FTIR) is a widely uesd vibrational spectroscopy technique, which enables indirect characterization of VOCs by providing spectroscopic information on the overall properties of the samples through measurements within the ranges of 4000–400 cm^−1^ and the fundamental frequency vibrations of the molecular groups (Gao et al., 2024). The two-dimensional correlation spectroscopy (2DCOS) derived from FTIR data exhibits superior discrimination of sample compositional differences versus the conventional one-dimensional FTIR (1D-FTIR) spectroscopy (Noda, 2018). Zheng et al. (2025) amplified the differences in VOCs in *Boletus. bainiugan* at different drying temperatures by FTIR-2DCOS for further characterization. An et al. (2024) employed FTIR-2DCOS and Raman-2DCOS data to further characterize the starch degradation process to analyze the continuous variations in starch composition. FTIR-2DCOS has proven to be an effective analytical tool for characterizing and differentiating chemical composition in samples. As a rapid, eco-friendly, and straightforward technique, FTIR spectroscopy coupled with chemometric analysis has been successfully applied to the qualitative and quantitative analysis of herbs and foods (Deng et al., 2025b; Yuan et al., 2022). Zheng et al. (2025) described that the combination of FTIR spectroscopy and HS-SPME-GC–MS data to construct a partial least squares regression (PLSR) model successfully predicted the major VOCs in *Boletus bainiugan* (VIP > 1, relative odor activity value (rOAV) > 1, *p* < 0.05). Park et al. (2024) identified that FTIR data combined with GC–MS data to establish a PLSR model could effectively predict the VOCs in *Katsuobushi*, with the potential to assess the quality of *Katsuobushi*. A separate investigation successfully quantified perilla ketone and isoegomaketone in *Perillae Folium* VOCs by means of PLSR modeling of FT-NIR data supplemented by GC–MS, achieving residual prediction deviation (RPD) values of 3.76 and 2.59, respectively (Yu et al., 2024). In conclusion, the PLSR constructed by spectroscopic techniques and GC–MS data could effectively predict the VOCs levels. However, current research on *G. elata* predominantly focuses on the extraction and isolation of active ingredients and their related pharmacological properties (Su et al., 2023; Zhong et al., 2025), while studies on rapid quantification of key VOC indicators in *G. elata* across different geographic origins using FTIR technology remain limited.

The present study investigates the potential of FTIR spectroscopy coupled with chemometric analysis for both geographical origin authentication and key VOCs quantification in *G. elata*. The specific objectives were as follows: 1) to analyze VOCs of *G. elata* from different regions using HS-SPME-GC–MS and identify their major flavors of *G. elata*; 2) to obtain the FTIR data of *G. elata* from different regions and construct a qualitative discriminant model based on chemometric analysis; 3) to establish a PLSR-based quantitative model using the FTIR and HS-SPME-GC–MS data for predicting key VOCs contents of *G. elata* across different regions. A comprehensive analysis of *G. elata* VOCs from diverse origins using FTIR technology will provide insights into the differences in key VOCs and the dominant flavors of *G. elata* across different production regions. The present will establish a rapid, co-friendly and comprehensive method for VOCs quality assessment in *G. elata*, which could also be extended to other herbs, spices and foods.

## 2 Materials and methods

### 2.1 Materials and sample preparation

A total of 167 *G. elata* samples were collected in November 2023 from the following areas: YNZT (75 samples); Bijie, Guizhou Province (GZBJ, 46 samples); and Yichang, Hubei Province (HBYC, 46 samples). The fresh *G. elata* rhizomes were brushed to remove associated fungi and surface soil after digging. This study used two-year-old *G. elata* samples that were morphologically intact, oval in shape and uniform in size. The samples had a diameter of 3–5 cm, a length of 7–10 cm, and a single weight of 100–160 g, and exhibited no surface damage. The phenotypes of *G. elata* from different origins are shown in Fig. S2. Samples of *G. elata* from different origins were sliced separately and dried at 60 °C until they reached a constant weight. Subsequently, 20 mixed samples were collected from the YNZT origin, and 15 mixed samples each from GZBJ and HBYC origins, respectively. All samples were stored in an ultra-low-temperature refrigerator at −80 °C for subsequent VOCs analysis by HS-SPME-GC–MS. The remaining samples were pulverised using a pulveriser, passed through a 100-mesh sieve (GB/T6003.1-2012) and stored in labelled ziplock bags at room temperature for FTIR spectra acquisition (Deng et al., 2025b).

### 2.2 Volatile component detection of metabolites

#### 2.2.1 Sample pretreatment

The VOCs of *G. elata* samples from various sources were measured by Metware Metabolic Biotechnology Co., Ltd. (Wuhan, China) using an HS-SPME-GC–MS system (Deng et al., 2025b; Zheng et al., 2025). The process for the experiment was as follows: samples were removed from the −80 °C ultra-low-temperature refrigerator, ground using liquid nitrogen， and vortexed to mix; approximately 500 mg of the samples were weighed into head-space vial (Agilent, Palo Alto, CA, USA). Subsequently, 20 μL of an internal standard solution, 3-Hexanone-2,2,4,4-d4 (10 μg/mL, BioBioPha, Yunnan, China), was added to each headspace vial, and the addition of a saturated NaCl solution (26.47 %, China National Medicines Corporation Ltd., Beijing, China). Lastly, samples were extracted using the SPME automated solid-phase microextraction device (SPME Arrow, CTC Analytics AG, Zwingen, Switzerland) for GC–MS (8890-7000D, Agilent, Santa Clara, CA, USA) analysis (Zhang et al., 2025).

#### 2.2.2 HS-SPME analysis

The HS-SPME extraction procedure was as follows: the samples were shaken and equilibrated for 5 min at 60 °C, after which an SPME extraction needle (120 μm DVB/CWR/PDMS, Agilent, Santa Clara, CA, USA) was placed into head-space vial and extracted for 15 min. The extract was then desorbed at 250 °C for 5 min, and introduced into the GC–MS system for separation and identification. Before sampling, the fiber should be aged in a Fiber Conditioning Station at 250 °C for 5 min (the new fibers should be aged under the same conditions for 2 h before first use).

#### 2.2.3 GC–MS analysis

The GC–MS instrument was set up as follows: the DB-5MS capillary column (30 m × 0.25 mm × 0.25 μm; Agilent, Folsom, CA, USA) was used with high-purity helium (purity ≥99.999 %) as the carrier gas at a constant flow rate of 1.2 mL/min. The inlet port was maintained at 250 °C, and the injection was performed in splitless mode with a solvent delay of 3.5 min. The oven temperature program was as follows: initial temperature 40 °C for 3.5 min; then ramped to 100 °C at 10 °C/min. Then, ramped to 180 at 7 °C/min; and finally ramped to 280 °C at 25 °C/min for 5 min at the end of the run. The chromatograms of *G. elata* from different origins and integral diagrams of some VOCs chromatograms are shown in Fig. S4 and S5.

MS spectrometry detection was carried out using an electron bombardment ion source (EI) with an ion source temperature of 230 °C, quadrupole temperature of 150 °C, mass spectrometry interface temperature of 280 °C, 70 eV as electron energy. Detection was performed in selected ion (SIM) mode with narrow scanning of qualifying and quantifying ions/peaks. The MS spectrum of some VOCs is shown in Fig. S6. Quality Control (QC) samples were prepared from a mixture of samples to quantify the reproducibility of these analytical methods. One QC sample was included after every 10 assay samples analyzed in the experiment to assure stability in the measure analytical stability. The total ion flow diagrams (TIC) of different QC samples are shown in Fig. S3, and their good reproducibility verifies the high stability of the instrument, which provides an important guarantee for data reliability.

#### 2.2.4 Qualitative and quantitative analysis

During qualitative analysis using the Agilent MassHunter software, the deconvolution settings were configured as follows: The peak width was set to 20, sensitivity, resolution, and chromatographic peak shape were all adjusted to moderate, and the minimum matching factor was set to 70. The acquired mass spectrometry data were compared with the mass spectra of standard substances in the National Institute of Standards and Technology (NIST, version 14.0) library for substances identification, and the retention index (RI) of each volatile compound were determined. Employing an n-alkane (C_7_ – C_40_) blend as the reference, GC–MS analysis was conducted following identical chromatographic conditions. The RI values of the compounds were calculated using Eq. 1 and evaluated against documented RI values from the NIST database. The RI screening criteria were set as a deviation within ±30 of the reference value.

(1) $$\mathrm{RI}=100Z+\frac{100\;\left[\mathrm{TR}\;\left(x\right)-\mathrm{TR}\;\left(z\right)\right]}{\mathrm{TR}\;\left(z+1\right)-\mathrm{TR}\;\left(z\right)}$$

Where TR(x) represents the retention temperature of the component, TR (z) and TR (z + 1) represent the retention temperatures of n-alkanes with carbon numbers Z and Z + 1, respectively, where TR (z) < TR (x) < TR (z + 1).

Furthermore, literature reports were integrated to enhance the validation of qualitative results. Additionally, a specialized high-coverage plant volatile library was constructed, and a comprehensive SIM (cSIM) acquisition method was developed to achieve high sensitivity, high coverage, and accurate substance annotation (Yuan et al., 2024). During the detection process, if the retention time (RT) and characteristic fragments of a compound match the parameter settings, the compound is identified as the target substance (GB 23200.8-2016). The methodological principles have been well supported by relevant literature and, in conjunction with the national standard GB 23200.8-2016, substantiate the feasibility of the broad-target SIM-based qualitative and quantitative approach in this study (Yuan et al., 2024). Notably, the RI was incorporated as a critical parameter during library construction, and spot scanning was performed within the confidence interval (±30), thus eliminating the need for additional RI-based qualitative data.

For quantitative analysis, an appropriate compound standard was selected to ensure compliance with methodological requirements, and a known quantity of the standard was spiked into the sample. Both the analyte and the standard were subsequently analyzed in parallel. Based on relevant studies, isotopes are currently one of the most commonly used internal standard reagents. Therefore, we selected an isotope-based internal standard and used Eq. 2 to calculate the relative content of *V*OCs in the sample.

(2) $$X_{i}=\frac{V_{S}\times C_{s}}{m}\times \frac{I_{i}}{I_{s}}\times 10^{-3}$$

X_*i*_ represents the concentration of compound *i* in the sample to be analyzed (μg/g); V_s_ is the volume of internal standard added (μL); C_s_ is the concentration of internal standard (μg/mL); m is the amount of sample to be analyzed (g); I*s* is the peak area of the internal standard; I_*i*_ is the peak area of compound *i* in the sample to be analyzed.

### 2.3 rOAV analysis

The rOAV method, which integrates the sensory thresholds of the compounds, is used to determine the key flavor compounds in foods and can elucidate the contribution of each aroma compound to the overall aroma characteristics of the samples. The rOAV has been increasingly used to determine key flavor compounds in different foods. In general, rOAV ≥1 denotes that the compound contributes directly to the flavor present in the sample, while rOAV ≥100 denotes that the volatile component contributes immensely to the sample's aroma profile (Wang et al., 2025). The calculation is shown in Eq. 3:

(3) $$\mathrm{rOA}V_{i}=\frac{C_{i}}{T_{i}}$$

where rOAV_*i*_ is the relative odor activity value of compound *i*, C_*i*_ and T_*i*_ are the relative content and the Threshold of the compound (μg/mL).

### 2.4 FTIR acquisition and pre-processing

An attenuated total reflection FTIR spectroscopy system (ATR-FTIR, Thermo Fisher Scientific) was used to characterize the samples by FTIR spectroscopy. The spectral acquisition parameters were set as follows: the number of scanning accumulations was 64, the spectral resolution accuracy was 4 cm^−1^, and the wave number range covered the mid-infrared characteristic region of 4000–400 cm^−1^. To eliminate interference from ambient gases, blank background spectra were acquired at 30 min inter*v*als, and background subtraction was performed during sample spectral scanning. Each sample was acquired three times in parallel and the three raw spectral data were a*v*eraged spectra in the OMNIC software that comes with ATR-FTIR for further analysis.

Preprocessing techniques can reduce instrumental background noise, eliminate baseline drift, and resolve overlapping infrared bands, which is conducive to further improving the robustness of the model. This study examines a total of 5 different of preprocessing methods, including first-order derivative (1stDer) and second-order derivative (2ndDer) transformations, Savitzky-Golay (SG) smoothing algorithm based on an 11-point smoothing window and a 3rd-order polynomial, standard normal variable (SNV) and multivariate scattering correction (MSC) (Deng et al., 2025a; Li et al., 2025b).

### 2.5 2DCOS image dataset acquisition

The generalized 2DCOS image is enhanced with spectral resolution by introducing a perturbation factor to resolve overlapping signals (Noda, 2018). This method involves acquiring a discretized dynamic spectrum through external perturbation, which can be mathematically characterized as shown in Eq. (4). Here, the reference spectrum Y (*v*) is defined as the sample mean spectrum, t is the perturbation variable, and m is the number of spectral measurements.

(4) $$Y\left(v\right)=\left\{\begin{array}{c} Y\left(n,t_{1}\right)\\ Y\left(n,t_{2}\right)\\ .\\ .\\ .\\ Y\left(n,t_{m}\right) \end{array}\;\right)$$

The synchronized 2DCOS image (Φ (n₁, n₂)) can be obtained by calculating the covariance of the dynamic spectral vectors in different bands (n₁, n₂) (Eq. 5). It can intuitively reflect the characteristics of the spectral signal that changes in concert with the perturbation.

(5) $$\Phi \;\left(n_{1},n_{2}\right)=\frac{1}{m}\;f\;{\left(n_{1}\right)}^{T}\cdot f\;\left(n_{2}\right)$$

The experiment used the surf function of MATLAB R2023a to construct 128 × 128 pixel JPEG format images and applied the Kennard-Stone (K—S) algorithm to divide the 166 synchronized 2DCOS images into a training set (120 images), a test set (31 images), and an external validation set (15 images) in the ratio of 7:2:1. The residual neural network (ResNet) deep learning model is constructed using the obtained synchronized 2DCOS images as shown in Fig. S7.

### 2.6 Model establishment and evaluation

#### 2.6.1 Classification models

In this study, classical pattern recognition methods and modern chemometrics algorithms were combined to conduct a traceability study of the origin of *G. elata* samples. First, an unsupervised principal component analysis (PCA) model was constructed for exploratory data analysis. This method reduces dimensionality by projecting high-dimensional data onto the first few principal components and visualizing the clustering and grouping characteristics of the samples in the resulting low-dimensional space (Zheng et al., 2023). Subsequently, orthogonal partial least squares discriminant analysis (OPLS-DA), a common supervised model, was constructed to separate *G. elata* samples from different origins. The model inherits and develops the mathematical framework of the partial least squares (PLS) algorithm, and effectively addresses the complex covariance problem among high-dimensional variables in the classification task by constructing the latent variable space (Yu et al., 2024). The validation of the OPLS-DA model is based on three key performance indicators: 1) the explained variance (R^2^X), which is used to assess the modeling effectiveness of GC–MS/FTIR spectral data; 2) the discriminant variance (R^2^Y), which reflects the goodness of fit of the categorical variables; and 3) the cross-validated predictive power (Q^2^), which measures the generalization performance of the model. When the values of R^2^X, R^2^Y and Q^2^ are close to 1, it indicates that the model has optimal explanatory power and predictive accuracy. In addition, a support vector machine (SVM) model was constructed to extract the most effective information for classification. The model chose radial basis function (RBF) as the kernel function, which is characterized by a simple structure, high computational efficiency, and excellent adaptability to high-dimensional feature data (Jin et al., 2020; Yu et al., 2025). Two key parameters of the RBF kernel function are the penalty factor (*c*) and the kernel parameter (*g*). The combination of optimal or near-optimal parameter values for *c* and *g* determines the generalization performance of the model, thereby effectively avoiding the problems of underfitting or overfitting. Therefore, to achieve the parameter optimization objective, this study constructed optimized parameter combinations (*c*, *g*) using grid search (GS), the range of parameter (*c*, *g*) for the GS algorithm is set to be (2^−10^,2^10^); for the genetic algorithm (GA) algorithm, parameter *c* varies in the range of (0,100) and *g* varies in (0,1000); for the gray wolf optimizer (GWO) algorithm, both *c* and *g* vary in the range of (1 × 10^−5^, 1000). Through determining the optimal parameter combination, this study aiming to construct SVM discriminative models with strong generalization ability. It is worth noting that for the OPLS-DA and SVM models constructed based on spectral data in this paper, the samples were divided into a training set (120 samples) and a test set (46 samples) at a ratio of 7:3 using the K—S algorithm.

ResNet, as an efficient convolutional neural network architecture, effectively solves the problem of gradient vanishing/exploding in the traditional CNN model by introducing the residual connectivity mechanism, and exhibits excellent performance characteristics in spectral image analysis applications (Dong et al., 2023; Li et al., 2025b). The ResNet architecture constructed in this study is based on the team's previous research (Dong et al., 2023). To effectively mitigate the common overfitting problem in deep neural networks, a shallow ResNet architecture containing 12 residual blocks was adopted by optimizing the complexity of the network structure. The hyperparameters of this model are systematically tuned by setting the initial learning rate to 0.01 and introducing L2 regularization (weight decay coefficient λ = 0.0001) to enhance the generalization performance of the model while ensuring its expressive ability.

The constructed SVM and ResNet models are evaluated using accuracy (ACC), F_1_ score, precision (P) and recall (R) and to evaluate the performance of the models. Its specific formula can be referred to in the study of Gao et al. (2024).

#### 2.6.2 Quantitative model

A PLSR model was constructed to quantitatively predict the levels of key VOCs in *G. elata* samples. The algorithm decomposes the spectral (X, FTIR data) and content (Y, relative content of VOCs) matrices by iteratively calculating latent variables (LVs). This maximizes the covariance between X and Y while effectively filtering out orthogonal noise interference in the original data, thereby establishing the relevance between spectral features and indicators (Fakhlaei et al., 2025; Yuan et al., 2022). The core advantage of PLSR, as the most commonly used multivariate correction method in spectral analysis, lies in its ability to maximize the extraction of effective spectral information, thereby significantly improving the modeling effect. Before modeling, the K—S algorithm was used to divide the spectral data of *G. elata* from different origins and their corresponding GC–MS data (a total of 50 samples) into calibration and prediction sets at a ratio of 2:1.

The performance of the model is evaluated by following key index parameters: the calibration and prediction set coefficients of determination (R^2^_C_ and R^2^_P_, respectively), the calibration and prediction set root-mean-square errors (RMSE_C_ and RMSE_P_, respectively), and the calibration and prediction set RPD (RPD_C_ and RPD_P_, respectively). The corresponding formulas can be found in the study by Zhu et al. (2025). Generally, high R^2^_C_ and R^2^_P_ values (greater than 0.8) and low RMSE_C_ and RMSE_P_ values indicate good model performance. The size of the RPD value directly reflects the PLSR model's overall predictive performance. The larger the RPD value, the better the model's performance (Bhuiyan et al., 2024; Fakhlaei et al., 2025). When RPD > 2.0, the model is considered to have strong predictive ability and to be suitable for production practice (Deng et al., 2025b).

### 2.7 Statistical analysis

The K—S algorithm, 2DCOS images, and SVM models were all calculated using MATLAB R2023a (The MathWorks, Inc., Natick, MA, USA). Spectral preprocessing, OPLS-DA, and PLSR models were completed using SIMCA 14.1 (Sartorius Stedim Data Analytics Umeå, AB, Sweden). PCA analysis and Venn diagrams were performed using Origin Pro 9.0 (OriginLab Corporation, Northampton, MA, USA). The ResNet model and key VOCs molecular structures were constructed using Spyder (Anaconda3 2023.07 distribution, Anaconda Inc., Austin, TX, USA) and ChemBioDraw Ultra 14.0 (PerkinElmer Informatics, Waltham, MA, USA), respectively.

## 3 Results and discussion

### 3.1 HS-SPME-GC–MS analysis

Based on the GC–MS detection platform and Metware database, a total of 538 VOCs were identified in *G. elata* samples from three production regions of YNZT, GZBJ and HBYC (Fig. S8a). These VOCs included 116 terpenoids, 46 hydrocarbons, 77 ketones, 50 heterocyclic compounds, 75 esters, 17 amines, 50 alcohols, 10 aromatics, 22 phenols, and 13 nitrogenous compounds, 9 ethers, 25 aldehydes, 23 acids, 2 Sulfur compounds and 3 halogenated hydrocarbons. Information on metabolite numbers, integral values and corresponding metabolites is provided in Supplementary Material 2. In comparison with previous studies, our study identified the most diverse range of VOCs in *G. elata*. The aroma of *G. elata* is primarily determined by the composition and relative content of its VOCs, which are significantly affected by factors such as variety, origin, stage of maturity and processing methods (Wang et al., 2025; Li, Zhang, Nan and Yuan, 2025; Li et al., 2025). Previous studies employing electronic nose (e-nose), HS-SPME-GC–MS, and HS-GC-IMS techniques have revealed that alcohols and ketones are the main contributors to the aroma of *G. elata* from different origins (Wang et al., 2025). In a recent study, gas chromatography quadrupole-time of flight mass spectrometry (GC-QTOF-MS) was employed to analyze the VOCs of *G. elata* at various stages of growth, identifying 87 volatiles with characteristic aromas including aromatic hydrocarbons, terpenes, aldehydes and esters (Li, Zhang, Nan and Yuan, 2025). Another study employed GC-IMS technology to identify 58 compounds, including aldehydes, hydrocarbons, alcohols and ketones, present in *G. elata* that had been processed using various methods (Li et al., 2025). Semi-quantitative analysis of VOCs suggested that there were significant differences among samples from different production regions, with GZBJ samples showing relatively lower VOCs levels (Fig. S8a). This phenomenon may be attributed to the high-humidity environment in GZBJ, which potentially suppresses the release rate of plant VOCs and possibly affects the activity of specific oxidases (e.g., the LOX pathway), thereby leading to a reduced production of aldehydes, alcohols, and lipids (Dicke & Loreto, 2010).

**Fig. 1:**
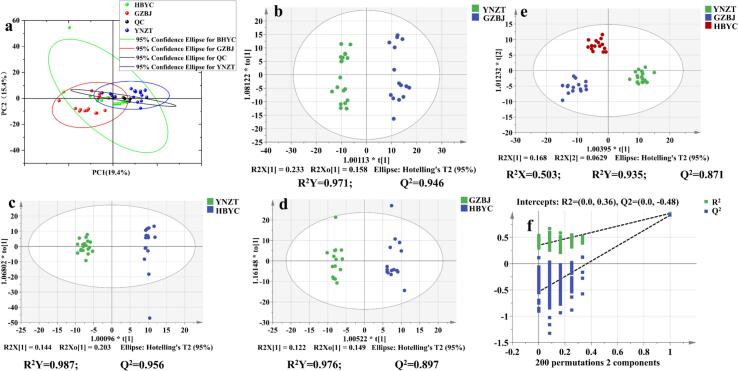
Multivariate statistics of *G. elata* from different origins based on GC–MS data. (a) PCA score plots; (b) OPLS-DA score plots for YNZT vs GZBJ; (c) OPLS-DA score plots for YNZT vs HBYC; (d) OPLS-DA score plots for GZBJ vs HBYC; (e) OPLS-DA score plots based on volatiles; and (f) validation plots for 200 permutation tests of the OPLS-DA model. YNZT: Zhaotong, Yunnan Province; GZBJ: Bijie City, Guizhou Province; HBYC: Yichang City, Hubei Province.

To explore these differences, unsupervised PCA and supervised OPLS-DA models were established and the volatiles detected by GC–MS were used as independent variables and *G. elata* samples from different origins as dependent variables. The PCA results described that the QC samples were clustered in the middle of the *G. elata* samples, which indicated the excellent reproducibility and stability of the GC–MS data (Deng et al., 2025b), whereas PCA failed to clearly distinguish the three groups of volatile metabolites (Fig. 1a). Thus, based on the VOCs data from the three production areas, the supervised OPLS-DA model was employed for statistical analysis. The results displayed that the model could achieve satisfactory separation of *G. elata* samples from different origins, with R^2^X = 0.503, R^2^Y = 0.935, and Q^2^ = 0.871, indicating the potent model fit and prediction ability (Fig. 1e). A 200 permutation test showed green and blue data points on the right side higher than those on the left, with R^2^ = 0.36, which is less than 0.4, and Q^2^ = −0.48, which is less than 0.05 (Fig. 1f). These findings indicated that there is no overfitting of the model and verified the model validity and predictability. To further identify the major VOCs of *G. elata*, Zheng et al. (2025) identified that VOCs contributed to the aroma of samples when OAV >1 (Zheng et al., 2025). As illustrated in Table S1, 72 components were identified as contributing to the aroma of *G. elata* based on the rOAVs calculation formula. These were mainly concentrated in aldehydes, terpenoids, alcohols and ketones, and exhibited the characteristic flavor peaks of *G. elata,* which are sweet, fruity, green, fatty and nutty. These results are consistent with those of previous studies (Wang et al., 2025; Li, Zhang, Nan and Yuan, 2025).

**Fig. 2:**
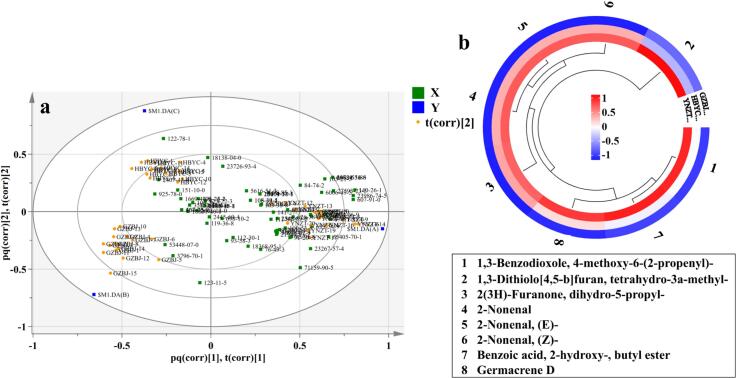
(a) HS-SPME-GC–MS/MS data biplot; (b) The circular heat map of hierarchical cluster analysis of metabolites of 8 VOCs in different *G. elata* provenances (colors from red to blue represent high to low concentrations). (For interpretation of the references to colour in this figure legend, the reader is referred to the web version of this article.)

In addition, the correlation between different origins and various VOCs was investigated (Fig. 2a). The correlation strength between the samples and VOCs was characterized by their spatial proximity, with a shorter distance indicating stronger correlation. The results revealed that most VOCs were highly correlated with the YNZT samples, including benzenemethanol,.alpha.-methyl-;2,6-Octadienal; 3,7-dimethyl-, (*E*)-; 2-Hexenal, (E); 2-Hexenal; 2-Pentanol, acetate; benzenemethanethiol; 1,3-Dithiolo[4,5-*b*]furan, tetrahydro-3a-methyl-; 2-Propenoic acid, 3-phenyl-, methyl ester; pyrazine, 2-ethyl- 3,5-dimethyl-; 2-Decanone; 3,4-Dimethyl-1,2-cyclopentadione and others. The substances were highly correlated with the GZBJ samples including 2-Undecenal, *E*- and 5,9-Undecadien-2-one, 6,10-dimethyl-, (*E*)-. Additionally, 2(5H)-Furanone, 5-ethyl-, and Benzene, 1,3-dimethoxy- were strongly correlated with the HBYC samples. These results demonstrate that YNZT *G. elata* presents significantly greater flavor richness and intensity than GZBJ and HBYC. This difference may be closely related to YNZT's unique geographical environment (Zheng et al., 2023). The area's specific climate factors (temperature, humidity and sunlight) and soil characteristics may create more favourable ecological conditions for the synthesis and accumulation of secondary metabolites in *G. elata*, thereby promoting the formation efficiency of volatile flavor compounds. In order to explore the differences between the groups, the samples from three origins were analyzed through pairwise comparison using OPLS-DA. The results demonstrated the most distinct separation effect between YNZT and HBYC groups, with an R^2^Y value of 0.987 and a Q^2^ value of 0.956 (Fig. 1c). This was followed by the YNZT and GZBJ regions with parameter values of 0.971 and 0.946 for R^2^Y and Q^2^, respectively (Fig. 1b). In contrast, the differences between the GZBJ and HBYC regions were smallest, with an R^2^Y of 0.976 and a Q^2^ of 0.879 (Fig. 1d). These data suggest that the VOCs of *G. elata* is closely related to its geographic origin, with production regions in closer proximity exhibiting greater compositional similarity. To identify the major differential VOCs in the different producing regions (YNZT, GZBJ and HBYC provinces), the screening criteria for the raw data were presented as follows: inter-subgroup VIPs >1, OAVs >1, and *p*-values ≤0.05. Comparative analysis of differential volatile metabolites among the subgroups revealed eight common VOCs shared across all three regions, including 2-Nonenal; 2- Nonenal, (E)-; 2-Nonenal, (*Z*)-; 2(3H)-Furanone, dihydro-5-propyl-; Benzoic acid, 2-hydroxy-, butyl ester; 1,3-Benzodioxole, 4- methoxy-6-(2-propenyl)-; Germacrene D; 1,3-Dithiolo[4,5-*b*]furan, tetrahydro-3a-methyl- (Fig. S8b, Fig. S9). The identification results indicate significant interregional differences in VOC content, with *G. elata* from YNZT exhibiting the highest overall VOC levels. (Table 1, Fig. 2b). Hence, VOCs in *G. elata* are influenced by geography, and the flavor profiles exhibit distinct origin-specific patterns, with eight crucial VOCs identified as key flavor-differentiating variables in *G. elata* of the geographical region. In conclusion, GC–MS coupled with OPLS-DA can effectively discriminate *G. elata* samples from different production origins, establishing its application for origin tracing. However, due to the limitations of the traditional GC–MS method, such as prolonged analysis time and operational complexity, rapid identification of *G. elata* production region and prediction of major VOCs has become the focus of current research. Therefore, we will next combine FTIR with chemometrics to improve the identification ability and quality evaluation of *G. elata* from different origins.

**Table 1:** The main differential volatile compounds of *G. elata* in different production areas (rOAV ≥1, VIP > 1.0, *P* < 0.05).

Index	Compounds	Category	Formula	CAS	NIST_RI	RT (min)	Odor	P	ROVs			Concentration(ug/g)		
									YNZT	GZBJ	HBYC	YNZT	GZBJ	HBYC
KMW0343*263	2-Nonenal	Aldehyde	C_9_H_16_O	2463-53-8	1161	12.499	Fatty, green, waxy, cucumber, melon	1.19 × 10^−9^	4685.15	2244.7	3719	0.47 ± 0.06a	0.22 ± 0.15c	0.37 ± 0.04b
KMW0338*263	2-Nonenal, (*E*)-	Aldehyde	C_9_H_16_O	18,829–56-6	1161	12.499	Fatty, green, cucumber, aldehydic, citrus	1.19 × 10^−9^	5856.44	2805.9	4649	0.47 ± 0.06a	0.22 ± 0.15c	0.37 ± 0.04b
KMW0326*263	2-Nonenal, (*Z*)-	Aldehyde	C_9_H_16_O	60,784–31-8	1148	12.499	Orris, fatty, waxy, cucumber	1.19 × 10^−9^	104.11	49.88	82.65	0.47 ± 0.06a	0.22 ± 0.15c	0.37 ± 0.04b
KMW0317	2(3H)-Furanone, dihydro-5-propyl-	Ester	C_7_H_12_O_2_	105–21-5	1156	12.498	Sweet, coconut, nutty, caramel, tonka, hay	1.77 × 10^−7^	1.63	0.83	1.32	0.65 ± 0.09a	0.33 ± 0.21c	0.53 ± 0.08b
D847*421	Benzoic acid, 2-hydroxy-, butyl ester	Ester	C_11_H_14_O_3_	2052-14-4	1469	18.240	Harsh, ethyl benzoate, clover	3.00 × 10^−6^	18.59	12.94	14.14	0.09 ± 0.01a	0.06 ± 0.01b	0.07 ± 0.01b
KMW0612	1,3-Benzodioxole, 4-methoxy-6-(2-propenyl)-	Ether	C_11_H_12_O_3_	607–91-0	1520	18.642	Spicy, warm, balsamic, woody	5.67 × 10^−11^	4.48	2.12	2.96	0.39 ± 0.08a	0.19 ± 0.06c	0.26 ± 0.05b
KMW0603*061	Germacrene D	Terpenoids	C_15_H_24_	23,986–74-5	1481	18.249	Woody, spice	2.11 × 10^−7^	107.1	69.83	83.66	0.13 ± 0.01b	0.08 ± 0.02ab	0.1 ± 0.02a
QWMW0245	1,3-Dithiolo[4,5-*b*]furan, tetrahydro-3a-methyl-	Heterocyclic compound	C_6_H_10_OS_2_	67,411–25-0	1245	13.877	Boiled, milky, chicken, cooked beef, rubbery, sulfury, thiamin	4.60 × 10^−5^	16.08	9.01	9.71	0.1 ± 0.04a	0.05 ± 0.01b	0.06 ± 0.01b

Means with different letters (a-c) in the same row are significantly different (*p* < 0.05).

### 3.2 FTIR spectral profiling

**Fig. 3:**
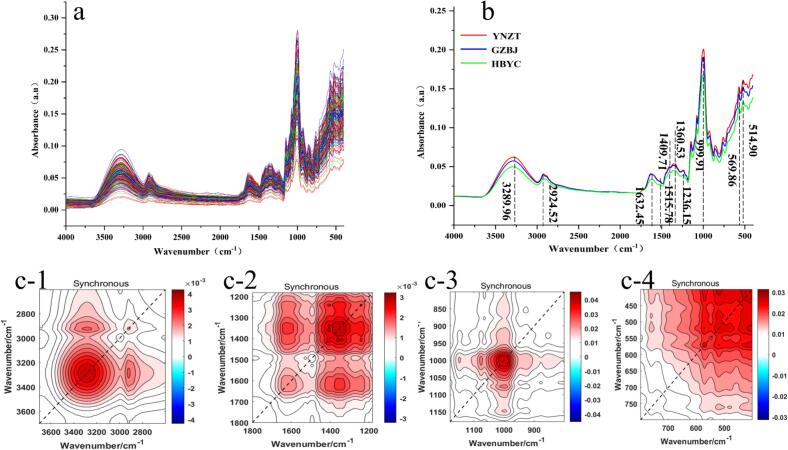
FTIR spectra of *G. elata*. (a) FTIR raw spectra, (b) FTIR averaged spectra; (c) synchronous 2DCOS images for spectral band (c-1) 3700–2600 cm^−1^, (c-2) 1800–1180 cm^−1^, (c-3) 1180–800 cm^−1^, (c-4) 800–400 cm^−1^ (In the synchronous spectra, the red/white regions (positive correlation) indicate a simultaneous change (stronger or weaker) in the absorption bands, while the blue regions (negative correlation) do the opposite). (For interpretation of the references to colour in this figure legend, the reader is referred to the web version of this article.)

**Fig. 4:**
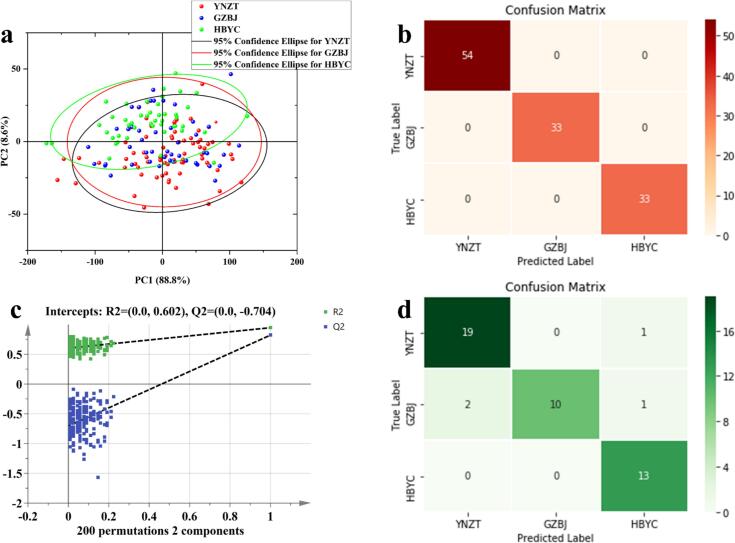
Multivariate statistics of *G. elata* from different provenances based on FTIR spectral data. (a) Plot of PCA scores based on the original spectra; (b) Plot of confusion matrix of OPLS-DA training set based on 1stDer preprocessing; (c) Plot of validation of the OPLS-DA model with 200 times of permutation tests; (d) The confusion matrix of the OPLS-DA test set is based on 1stDer preprocessing. YNZT: Zhaotong, Yunnan Province; GZBJ: Bijie City, Guizhou Province; HBYC: Yichang City, Hubei Province.

It can be seen the raw and averaged FTIR spectra of distinct origins of *G. elata* (Fig. 3a, b). The peak shapes, peak positions, peak widths and fluctuation trends of the FTIR spectra of *G. elata* from different origins were similar, possibly due to their similar chemical compositions. The FTIR broadband range was primarily concentrated around the 3700–2600 and 1800–400 cm^−1^ regions. The 3700–2600 cm^−1^ region was caused by C—H, N—H and O—H vibrations from alcohols and phenolic acids, peaked at 3289.96 cm^−1^ (Yuan et al., 2022). The C—H stretching vibrations of fatty acids and polyols caused absorption peaks in the vicinity of 2924.52 cm^−1^ (Zheng et al., 2025). Furthermore, most of the absorption peaks were concentrated within the 1800–400 cm^−1^ region. The 1752–1485 cm^−1^ region was associated with C—C vibrations in the aromatic rings of alcohols and terpenes, peaking at 1632.45 cm^−1^ (Fan et al., 2023). In the amide II spectral region, the absorption band at 1515.78 cm^−1^ arises from the combined contributions of C—N bond stretching and N—H group deformation vibrations (Bhuiyan et al., 2024; Deng et al., 2025a). There are lots of peaks at 1488–1180 cm^−1^ (1409.71 and 1360.53 cm^−1^) which are linked to C—H bending and stretching of C=O groups, and these come from ketones and aromatic compounds, among others (Deng et al., 2025a; Fakhlaei et al., 2025). In addition, the characteristic peak at 1236.15 cm^−1^ belongs to the C—O vibration associated with alcohols (Fan et al., 2023). The peak at 999.91 cm^−1^ is associated with C-O-H bending and C—C stretching vibrations, which mainly originate from carbohydrates, aromatic compounds, and polysaccharides (Deng et al., 2025a; Zheng et al., 2025). Several absorption peaks (at 569.86 and 514.90 cm^−1^) were also observed in the 600–400 cm^−1^ band. These are mainly associated with out-of-plane bending vibrations of C—H groups, O—H bending vibrations and -HC=CH- (trans) (Bhuiyan et al., 2024; Deng et al., 2025a). It is worth noting that Bhuiyan et al. (2024) indicated that the corresponding concentrations/amounts of compounds in a sample can be reflected by the relative absorbance values. Overall, the FTIR spectra showed that the YNZT sample had a higher average absorbance than the GZBJ and HBYC samples. This may be due to the YNZT sample having a relatively high content of chemical components. This result is consistent with those exhibited by GC–MS. 2DCOS-FTIR has a higher spectral resolution than 1D-FTIR. It amplifies differences in the chemical composition of *G. elata* from different origins and can solve the problem of hidden chemical information in 1D-FTIR spectra caused by many spectral overlaps (Noda, 2018). The intensity of the dynamic response of the same functional group under different perturbation conditions creates diagonal correlation peaks, known as autocorrelation peaks. Cross peaks mainly reflect the correlation between dynamic responses of different functional groups (or vibrational modes) under perturbation conditions. Based on the 1D-FTIR results, the FTIR spectrum was divided into four regions: 3700–2600, 1800–1180, 1180–800, and 800–400 cm^−1^. Fig. 3c shows the synchronous 2DCOS images. The chemical information was amplified at 3289.96 and 2924.52 cm^−1^, which is associated with alcohols, phenolic acids and fatty acids (Fig. 3c-1). Strong and broad autocorrelation peaks were mainly associated with alcohols, terpenes, proteins and ketones at 1632.45, 1515.7 and 1360.53 cm^−1^ (Fig. 3c-2). Additionally, broad and strong autocorrelation peaks appeared at 999.91 cm^−1^ in the 1180–800 cm^−1^ spectra, primarily associated with carbohydrates, aromatic compounds and polysaccharides (Fig. 3c-3). At 600 to 400 cm^−1^ in the spectrum, strong autocorrelation peaks appeared at 569.86 and 514.90 cm^−1^, mainly related to *G. elata* polysaccharides (Fig. 3c-4). In summary, averaged FTIR and 2DCOS spectra can effectively characterize the features and chemical composition differences of *G. elata* samples from different origins (Fig. 3 and Fig. S7). However, it is difficult to identify samples from distinct origins based on spectral characterization alone. It is necessary to further combine chemometrics to distinguish between origins.

### 3.3 PCA analysis

Based on the FTIR spectral data of *G. elata* from different production areas, this study first constructed an unsupervised PCA model to preliminarily explore the geographic origin characteristics of the samples and evaluate differences among samples from various regions. The first two principal components (PC1 = 88.8 % and PC2 = 8.6 %) cumulatively explained 97.4 % of the total variance in the variance of the original data (Fig. 4a). However, the results revealed substantial overlap in PCA space among samples from each origin, indicating that the model failed to effectively differentiate geographic sources (Li et al., 2025a; Zheng et al., 2023). Notably, YNZT and HBYC samples exhibited a tendency toward separation along PC1, which aligns with the greater spectral differences between these two regions compared to GZBJ in Fig. 3b. These findings highlight the limitations of PCA in origin discrimination and underscore the need for further in-depth analysis using supervised chemometric approaches.

### 3.4 OPLS-DA results analysis

Here, the effects of 5 spectral-preprocessing methods on the performance of the OPLS-DA model were systematically examined. As shown in Table S2, preprocessing strongly influenced on key model parameters (R^2^X, R^2^Y, Q^2^, and classification accuracy). The model built on 1stDer preprocessing exhibited optimal performance: R^2^X = 0.711, R^2^Y = 0.939, Q^2^ = 0.760, RMSE_CV_ = 0.210, RMSE_P_ = 0.241, with training- and test-set classification accuracies of 100 % and 91.30 %, respectively (Fig. 4b, d). These results indicate that the constructed 1stDer-OPLS-DA model possesses strong explanatory power and excellent model fit. This indicates that 1stDer is an effective pre-processing method for *G. elata* spectra from different origins, as it can improve the resolution of overlapping signals and correct baseline drift (Deng et al., 2025a; Li et al., 2025b). In addition, a 200 permutation test of the 1stDer-OPLS-DA model yielded Q^2^ value of −0.704 (below the 0.05 critical threshold), and the distribution of permuted data points on the right side (green and blue) were significantly exceeded that on the left side (Fig. 4d), confirming the model's lack of overfitting and robust validity (Li et al., 2025a). This demonstrates that FTIR spectral information can be used for initial geographical tracking of *G. elata*. In conclusion, the 1stDer-OPLS-DA model showed excellent performance (R^2^Y > 0.9, Q^2^ > 0.7) in identifying the origins of *G. elata*, offering a reliable tool for geographic traceability. To further improve the accuracy of geographic traceability, a 1stDer-based data matrix will be constructed in the subsequent analysis, followed by the application of machine learning algorithms to develop a more accurate identification model.

3.5 SVM results analysis.

**Fig. 5:**
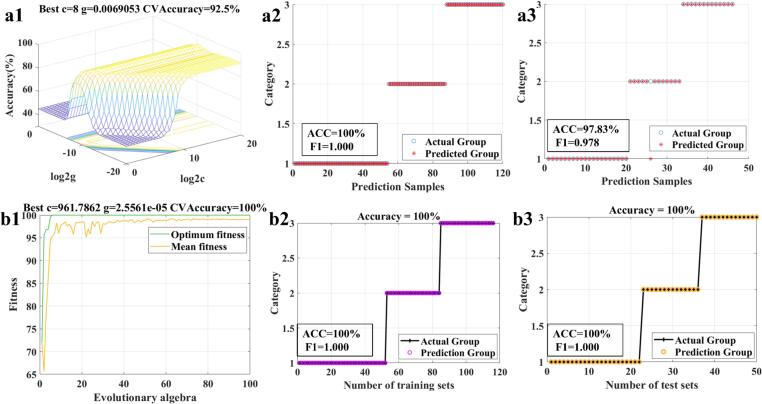
(a) 1stDer-GS-SVM model; (b) 1stDer-GWO-SVM model; (a1, b1) Optimal parameter optimization for (c, g) values; (a2, b2) Classification of training set; (c3, d3) Classification of test set.

To improve predictive accuracy and verify reliability, an SVM model was constructed for efficient classification of *G. elata* provenances based on FTIR spectra. GS, GA, and GWO were used to optimize the key hyperparameters, *c* and *g*, ensuring model robustness. Hyperparameters outside the optimal range or close to boundary values can compromise generalizability, leading to underfitting or overfitting (Yu et al., 2025). As shown in Table 2, using the full-wavelength spectral data matrix with 1stDer preprocessing, the GS-SVM model achieved 100 % accuracy and an F_1_ score of 1.000 on the training set, and 97.83 % accuracy with an F_1_ score of 0.979 on the test set (Fig. 5a), demonstrating excellent discriminative ability and stability. It is worth noting that the g-value of 1stDer-GA-SVM is 0.00095368, close to 0, indicating underfitting of the model (Table 2). The above results suggest that GS effectively identifies the optimal combination of hyperparameters through global optimisation, thus achieving a balance between high classification accuracy and generalization ability. By contrast, GA appears to be trapped in a local optimum during the optimisation process, failing to identify the optimal combination of the SVM parameters set.

**Table 2:** The classification results of the SVM model.

Model	Training sets				Test sets				c	g
	F_1_	P	R	ACC	F_1_	P	R	ACC		
**1stDer-GS-SVM**	**1.000**	**1.000**	**1.000**	**100 %**	**0.979**	**0.984**	**0.974**	**97.83 %**	**8**	**0.0069053**
1stDer-VIP-GS-SVM	1.000	1.000	1.000	100 %	0.931	0.94	0.923	93.48 %	64	0.0017263
1stDer-GWO-SVM	1.000	1.000	1.000	100 %	0.974	0.974	0.974	95.65 %	713.4663	0.000022828
**1stDer-VIP-GWO-SVM**	**1.000**	**1.000**	**1.000**	**100 %**	**1.000**	**1.000**	**1.000**	**100 %**	**961.7862**	**0.0000256**
1stDer-GA-SVM	0.724	0.742	0.708	71.76 %	1.000	1.000	1.000	100 %	16.0675	0.00095368
1stDer-VIP-GA-SVM	0.7558	0.7542	0.7574	75.83 %	1.000	1.000	1.000	100 %	71.5084	0.00095368

The VIP > 1 criterion was then applied to screen spectral features reducing redundancy and multicollinearity. As shown in Table 2, the performance of the GS-SVM model declined after VIP-based screening, likely because certain discriminative variables were removed, diminishing predictive power (Yu et al., 2025). However, the 1stDer-VIP-GWO-SVM model improved the accuracy and precision of the prediction performance while significantly reducing the number of variables, yielding a simplified model. The model achieved perfect classification of *G. elata* samples from three origins, with 100 % accuracy and an F_1_ score of 1.000 on both the training and test sets, demonstrating excellent classification accuracy and stability (Fig. 5b). Previous studies have reported that the GWO-SVM model, combined with mid-level data fusion is suitable for geographic traceability of the geography of *yam* in Hebei, with a prediction accuracy of 100 % and an F_1_ score of 1.000 for both the training and test sets (Gao et al., 2024). Similarly, SVM models based on feature extraction techniques have demonstrated excellent recognition accuracy for the classification of *Tartary buckwheat* samples (Yu et al., 2025). In summary, VIP variable screening accurately identifies feature wavenumbers that are significantly related to origin, while eliminating irrelevant variables. 1stDer-VIP-GWO-SVM model significantly improves the modeling efficiency without sacrificing classification accuracy. It is confirmed that the parameter-optimized SVM exhibits excellent performance in small-sample linear classification problems, and its discriminative effect is significantly better than that of the OPLS-DA model. The algorithm maintains strong generalization ability under limited sample conditions, ensures the reliability of the prediction results, and achieves the global optimal solution. Moreover, the classification performance of this model is competitive with or superior to results from other machine learning algorithms applied to rapid species identification and origin traceability (Li et al., 2025a; Yu et al., 2024). However, it should be noted that machine learning models usually require complex preprocessing and feature extraction processes to achieve high robustness (Gao et al., 2024; Yu et al., 2025). Therefore, future research will focus on achieving accurate identification of *G. elata* origins without reliance on elaborate preprocessing, as discussed in the following sections.

### 3.5 ResNet results analysis

The ResNet model provides excellent recognition performance for 2DCOS images generated from raw spectra, with the significant advantage of eliminating the need for complex preprocessing and feature extraction. Zheng et al. (2024) constructed a ResNet model based on raw FT-NIR spectral data of *Boletus bainiugan* and confirmed that approach enables rapid and accurate classification of *Boletus* species and origins. Li et al. (2025b) reported that their 3DCOS-ResNet model, built 2DCOS and three-dimensional correlation spectroscopy (3DCOS) images from NIR spectra of *G. elata*, achieved 100 % accuracy on the test set and 95.45 % accuracy on the external validation set. Based on these findings, the present study constructed a ResNet model synchronous 2DCOS images generated from raw FTIR spectra of *G. elata* from different origins to construct a ResNet recognition model. Model performance was evaluated in terms of classification accuracy, loss function, and external validation results. A well-trained ResNet model typically exhibits high accuracy (close to 1) and low cross-entropy loss (close to 0). The results of the model performance evaluation show that when the epoch reaches 18, the model reached 100 % accuracy on both training and test sets, with the loss value decreasing to 0.163, indicating excellent recognition accuracy and generalization performance (Fig. S10a). This advantage may be attributed to its residual structure, which enables the extraction of key features from 2DCOS images. Unlike traditional 1D spectral analysis, 2DCOS reveals the interactions between different functional groups through synchronous/asynchronous correlation peaks, while ResNet's deep convolutions adaptively learn the associated spatial patterns. In the external validation set, all 15 samples were correctly classified with an accuracy and F_1_ of 100 % and 1.000, which fully confirmed the robust convergence characteristics and absence of overfitting in the model (Fig. S10b). However, it is important to acknowledge that the current dataset lacks samples from different cultivation years and extreme climatic conditions, which may result in an overestimation of model accuracy in actual applications. Overall, the 2DCOS-ResNet model demonstrated strong robustness and stability, offering a reliable technical solution for tracing the origins of *G. elata*.

### 3.6 PLSR model analysis

**Fig. 6:**
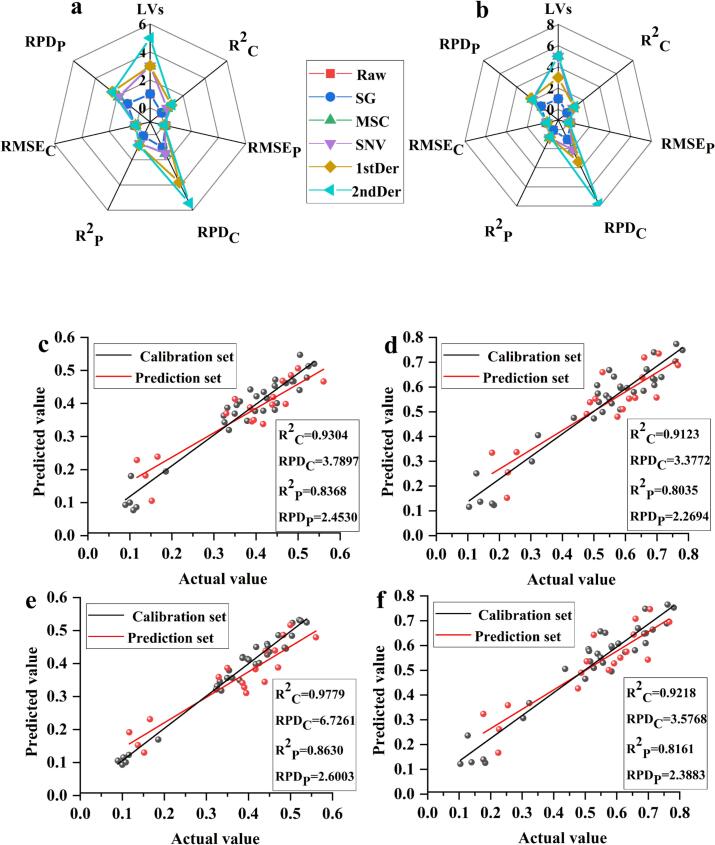
The main parameters of the (a) 2-Nonenal and (b) 2(3H)-Furanone, dihydro-5-propyl- PLSR models based on different pretreatment methods; Quantitative regression curves of (c) 2-Nonenal and (d) 2(3H)-Furanone, dihydro-5-propyl- in *G. elata* using 1stDer-PLSR model; Quantitative regression curves of (e) 2-Nonenal and (f) 2(3H)-Furanone, dihydro-5-propyl- in *G. elata* using 1stDer-VIP-PLSR model.

The analysis in Section 3.1 showed that the main flavor profiles of *G. elata* were sweet, fruity, green, fatty and nutty. Further analysis of 8 key differential volatile metabolites revealed that 2-Nonenal and its isomers ((*E*)-type and (*Z*)-type) as along with 2(3H)-Furanone, dihydro-5-propyl- were the primary contributors to these characteristic flavors, and thus serve as key markers of *G. elata* flavor quality. However, no studies have reported quantitative prediction of these major flavor components from different origins. Leveraging FTIR's capacity to capture comprehensive chemical information, we constructed a quantitative prediction model using PLSR to predict 2-Nonenal, its isomers, and 2(3H)-Furanone, dihydro-5-propyl- in *G. elata*, aiming to achieve a rapid and accurate assessment of the flavor quality of *G. elata* from different production areas. GC–MS analysis showed that 2-Nonenal and its isomers 2-Nonenal, (E)- and 2-Nonenal, (Z)- exhibited similar concentrations and chemical structures. Therefore, the PLSR model was established by correlating FTIR spectral data with relative content of 2-Nonenal and 2(3H)-Furanone, dihydro-5-propyl-. The effects of five different spectral pre-processing methods on the performance of the PLSR model were compared (Fig. 6a, b and Table S3). In evaluating 2-nonenal and 2(3H)-furanone, dihydro-5-propyl content in *G. elata*, the first Der-PLSR model showed excellent performance, with R^2^C values of 0.9304 and 0.9123, respectively, and R^2^P values of 0.8368 and 0.8035, respectively. (Fig. 6c, d) In addition, the RPD_P_ of the two models reached 2.4530 and 2.2694, respectively, with three optimal latent variables (LVs) and the lowest RMSE_P_. This is performance is attributed to the ability of 1stDer preprocessing to effectively correct baseline drift and enhance the resolution of overlapping signal features, making it easier for PLSR to capture the specific spectral bands of target compounds (Nagy et al., 2022). Moreover, the two optimal models have similar values of R^2^_C_ and R^2^_P_, a feature that effectively excludes the possibility of model overfitting or underfitting and confirms the predictive reliability of the models (Yu et al., 2024). To further optimize the model performance, the VIP method was applied to the 1stDer-preprocessed data to eliminate redundant spectral information. After the VIP screening, the model performance improved significantly (Table S4). For 2-Nonenal, the model R^2^_C_, R^2^_P_ and RPD_P_ reached 0.9779, 0.8630, and 2.6003, respectively, under the condition that the optimal LVs were equal to 4 (Fig. 6e). For 2(3H)-Furanone, dihydro-5-propyl-, the R^2^_C_, R^2^_P_ and RPD_P_ were 0.9218, 0.8161, and 2.3883, and the optimal LVs were 3 (Fig. 6f). In conclusion, the FTIR spectra of *G. elata* samples showed good linear correlations with the contents of 2-Nonenal and 2(3H)-Furanone, dihydro-5-propyl-. The application of the VIP method can effectively eliminate irrelevant spectral variables, select spectra sections closely related to the structure and content of VOCs, improve the predictive performance and generalization ability of the model, and is consistent with the findings of previous studies (Fan et al., 2023).

In recent years, various spectroscopic techniques combined with chemometric methods have advanced in the analysis of VOCs in food and Chinese herbal medicines (Yu et al., 2024; Zheng et al., 2025). Fan et al. (2023) innovatively integrated GC–MS, FT-NIR and FTIR spectroscopy with chemometrics to predict differential VOCs in Chinese herbal medicines, providing a valuable methodological reference for quality control. However, the rapid detection of VOCs of *G. elata*, an important edible and medicinal material, remains underexplored. This study demonstrates, for the first time that FTIR spectroscopy can be used to both trace the geographic origin and quantify key VOCs in *G. elata* rapidly and accurately, offering a novel technological tool for quality control of this rare medicinal material. These findings fill the research gap of rapid VOCs detection for *G. elata* and establish a methodological foundation for establishing its quality-standard system.

## 4 Conclusions

In this study, the accurate geographic traceability of *G. elata* from different origins and the rapid quantitative analysis of its key VOCs were successfully achieved for the first time by using FTIR spectroscopy and HS-SPME-GC–MS technology coupled with chemometrics methods. A total of 539 VOCs were identified by HS-SPME-GC–MS mainly characterized by sweet, fruity, green, fatty and nutty aromas, among which the flavor characteristics of *G. elata* from YNZT were significantly superior to those from GZBJ and HBYC. Based on the GC–MS data, OPLS-DA successfully differentiated the *G. elata* samples from different origins, and eight key differential VOCs were identified, including 2-Nonenal and 2(3H)-Furanone, dihydro-5-propyl-, etc. FTIR spectroscopy was further introduced to obtain spectral data of *G. elata* samples from different origins and enhance the efficiency of classification. The 1stDer-VIP-GWO-SVM classifier exhibiting the optimal discriminative ability of *G. elata* of different origins, achieving 100 % accuracy and an F_1_ score of 1.000 on both the training and test sets, In order to avoid complex preprocessing and feature extraction, this study innovatively transformed 1D-FTIR spectra into 2DCOS images and constructed a ResNet model, which achieved 100 % accuracy on the training set, test set and external validation set, showing excellent robustness and generalization. Subsequently, quantitative modeling analysis indicated that the 1stDer-VIP-PLSR model demonstrated excellent predictive capability for 2-Nonenal and 2(3H)-Furanone, dihydro-5-propyl-, achieving R^2^_P_ values greater than 0.8 and RPD_P_ values exceeding 2. Collectively, the comprehensive analytical method established in the present study provides a new and effective technique for both geographic traceability and quality control of key VOCs of *G. elata*. Moreover, it serves as a methodological reference for the quality evaluation of other food-medicinal plants and aromatic foods. Products. It should be noted that this study was limited to the three major production regions (YNZT, GZBJ and HBYC) due to sample size constraints. Given Tibet, Sichuan, Hunan and Shaanxi are also important habitat regions for *G. elata*, future studies should expand the sample collection and improve the analytical methods. Furthermore, future research is encouraged to integrate multi-source data such as hyperspectral, ultraviolet and near-infrared spectroscopy, with advanced VOCs analysis techniques (e.g., e-nose and HS-GC-IMS) and incorporate sensory evaluation methods. Such a multi-modal analytical framework will provide a more comprehensive and scientific explanation of the flavor quality of *G. elata*.

## CRediT authorship contribution statement

**Yingfeng Zhong:** Writing – original draft, Visualization, Data curation. **Jieqing Li:** Writing – original draft, Validation, Supervision. **Honggao Liu:** Software, Methodology, Funding acquisition, Formal analysis. **Yuanzhong Wang:** Resources, Project administration, Investigation, Conceptualization.

## Funding

This work was supported by the 10.13039/501100001809National Natural Science Foundation of China [GrantNumber: 82460746]; Yunnan Provincial Department of Education Scientific and Technological Innovation Team for Development and Utilization of *Gastrodia* Resources (Grant Number: 2024); Zhaotong “Xingzhao Talent Support Program” Team Project (Grant Number: 2023-3).

## Declaration of competing interest

The authors declare that they have no known competing financial interests or personal relationships that could have appeared to influence the work reported in this paper.

## Data Availability

The data that has been used is confidential.

## Appendix A Supplementary data

Supplementary material 1 Supplementary figures and tables for manuscripts. Supplementary material 1

Supplementary material 2 Volatile organic compounds raw data. Supplementary material 2

## References

[bb0005] An H., Ma Q., Zhang F., Zhai C., Sun J., Tang Y., Wang W. (2024). Insight into microstructure evolution during starch retrogradation by infrared and Raman spectroscopy combined with two-dimensional correlation spectroscopy analysis. Food Hydrocolloids.

[bb0010] Bhuiyan M.H.R., Liu L., Samaranayaka A., Ngadi M. (2024). Prediction of pea composites physicochemical traits and techno-functionalities using FTIR spectroscopy. LWT.

[bb0015] Deng G.M., Li J.Q., Liu H.G., Wang Y. (2025). Prediction of pyrazines and identification of flavor intensity in *boletus bainiugan* at different drying temperatures based on feature variables. Food Research International.

[bb0020] Deng G.M., Li J.Q., Liu H.G., Wang Y.Z. (2025). Rapid determination of geographical authenticity of *Gastrodia elata f. glauca* using Fourier transform infrared spectroscopy and deep learning. Food Control.

[bb0025] Dicke M., Loreto F. (2010). Induced plant volatiles: From genes to climate change. Trends in Plant Science.

[bb0030] Dong J.E., Li J.Q., Liu H.G., Wang Y.Z. (2023). Machine learning and deep learning based on the small FT-MIR dataset for fine-grained sampling site recognition of *boletus* tomentipes. Food Research International.

[bb0035] Du H., Liu C., Mei C., Zhan J. (2025). Progress in research on flavor compounds in *Gastrodia elata*. Journal of Food Bioactives.

[bb0040] Duan H., Zhou Y., Wang D., Yan W. (2023). Differences in volatile organic compounds in Rhizoma *Gastrodiae* (tian Ma) of different origins determined by HS-GC-IMS. Molecules.

[bb0045] Fakhlaei R., Babadi A.A., Ariffin N.M., Xiaobo Z. (2025). Development of FTIR-ATR spectra and PLS regression combination model for discrimination of pure and adulterated acacia honey. Food Control.

[bb0050] Fan Y., Bai X., Chen H., Yang X., Yang J., She Y., Fu H. (2023). A novel simultaneous quantitative method for differential volatile components in herbs based on combined near-infrared and mid-infrared spectroscopy. Food Chemistry.

[bb0055] Gao X., Dong W., Ying Z., Li G., Cheng Q., Zhao Z., Li W. (2024). Rapid discriminant analysis for the origin of specialty yam based on multispectral data fusion strategies. Food Chemistry.

[bib166] Gong M., Lai F., Chen J., Li X., Chen Y., He Y. (2024). Traditional uses, phytochemistry, pharmacology, applications, and quality control of Gastrodia elata Blume: A comprehensive review. Journal of Ethnopharmacology.

[bb0060] Jin G., Wang Y.J., Li L.Q., Shen S.S., Deng W.W., Zhang Z.Z., Ning J.M. (2020). Intelligent evaluation of black tea fermentation degree by FT-NIR and computer vision based on data fusion strategy. LWT.

[bb0065] Li G.Y., Li J.Q., Liu H.G., Wang Y. (2025). Geographic traceability of *Gastrodia elata* Blum based on combination of NIRS and Chemometrics. Food Chemistry.

[bb0070] Li G.Y., Li J.Q., Liu H.G., Wang Y.Z. (2025). Rapid and accurate identification of *Gastrodia elata* Blume species based on FTIR and NIR spectroscopy combined with chemometric methods. Talanta.

[bb0075] Li L., Zhang Y., Nan T., Yuan Y. (2025). GC-MS analysis of volatile organic compounds in *Gastrodia elata* during growth: Characterization of odor-active compounds associated with horse urine odor. Food Bioscience.

[bb0080] Li S., He X., Zhang X., Kong K.W., Xie J., Sun J., Wang Z. (2025). Integration of volatile and non-volatile metabolite profile, and in vitro digestion reveals the differences between different preparation methods on physico-chemical and biological properties of *Gastrodia elata*. Food Chemistry.

[bb0085] Ma M., Chen Z., Huang B., Chen X., Liu H., Peng Z., Dong P., Lu J., Wu D. (2024). Characterizing the key aroma compounds of barley malt from different origins using GC-E-nose, HS-SPME-GC-MS, and HS-GC-IMS. Food Bioscience.

[bb0090] Nagy M.M., Wang S., Farag M.A. (2022). Quality analysis and authentication of nutraceuticals using near IR (NIR) spectroscopy: A comprehensive review of novel trends and applications. Trends in Food Science & Technology.

[bb0095] Noda I. (2018). Two-dimensional correlation and codistribution spectroscopy (2DCOS and 2DCDS) analyses of time-dependent ATR IR spectra of d-glucose anomers undergoing mutarotation process in water. Spectrochimica Acta Part A, Molecular and Biomolecular Spectroscopy.

[bb0100] Park M., Yu J.Y., Ko J.A., Park H.J. (2024). Application of UV-vis-NIR and FTIR spectroscopy coupled with chemometrics for quality prediction of katsuobushi based on the number of smoking treatments. Food Chemistry.

[bb0105] Su Z.H., Yang Y.G., Chen S.Z., Tang Z.S., Xu H.B. (2023). The processing methods, phytochemistry and pharmacology of *Gastrodia elata* Bl.: A comprehensive review. Journal of Ethnopharmacology.

[bb0110] Wang L., Huang Y., Li Y., Chen Y., Chen G., Fang H., Ge Y. (2025). Analysis of key volatile components and their influencing factors in the production of indigenous *Gastrodia elata* in Guizhou. LWT.

[bb0115] Yu D.X., Qu C., Xu J.Y., Lu J.Y., Wu D.D., Wu Q.N. (2024). Rapid discrimination and quantification of chemotypes in Perillae folium using FT-NIR spectroscopy and GC–MS combined with chemometrics. Food Chemistry: X.

[bb0120] Yu Y., Chai Y., Yan Y., Li Z., Huang Y., Chen L., Dong H. (2025). Near-infrared spectroscopy combined with support vector machine for the identification of *Tartary buckwheat* (Fagopyrum tataricum (L.) Gaertn) adulteration using wavelength selection algorithms. Food Chemistry.

[bb0125] Yuan H., Cao G., Hou X., Huang M., Du P., Tan T., Zhang Y., Zhou H., Liu X., Liu L., Jiangfang Y., Li Y., Liu Z., Fang C., Zhao L., Fernie A.R., Luo J. (2022). Development of a widely targeted volatilomics method for profiling volatilomes in plants. Molecular Plant.

[bb0130] Yuan H., Jiang Y., Liu Z., Su R., Li Q., Fang C., Luo J. (2024). WTV2.0: A high-coverage plant volatilomics method with a comprehensive selective ion monitoring acquisition mode. Molecular Plant.

[bb0135] Zhang J.B., Wang B., Zhang Y.F., Wu Y., Li M.X., Gao T., Su L.L. (2024). E-eye and FT-NIR combined with multivariate algorithms to rapidly evaluate the dynamic changes in the quality of *Gastrodia elata* during steaming process. Food Chemistry.

[bb0140] Zhang Y., Gu M., Yang S., Fan W., Lin H., Jin S., Wang P., Ye N. (2025). Dynamic aroma characteristics of jasmine tea scented with single-petal jasmine “Bijian”: A comparative study with traditional double-petal jasmine. Food Chemistry.

[bb0145] Zheng C.M., Li J.Q., Liu H.G., Wang Y.Z. (2023). Data fusion of FT-NIR and ATR-FTIR spectra for accurate authentication of geographical indications for *Gastrodia elata* Blume. Food Bioscience.

[bb0150] Zheng C.M., Li J.Q., Liu H.G., Wang Y.Z. (2025). A new insight into the formation of special flavor during the drying process of boletes based on FTIR joint HS-SPME-GC-MS. LWT.

[bb0155] Zheng C.M., Liu H.G., Li J.Q., Wang Y. (2024). Identification and crude protein prediction of porcini mushrooms via deep learning-assisted FTIR fingerprinting. LWT.

[bb0160] Zhong Y., Li J., Liu H., Wang Y. (2025). The traditional uses, phytochemistry and pharmacology of *Gastrodia elata* Blume: A comprehensive review. Arabian Journal of Chemistry.

[bb0165] Zhu J., Zhu X., Yan B., Ren F., Chen B., Han Z., Yao X., He S., Liu H. (2025). Evaluation and categorization of various pea cultivars utilizing near-infrared spectroscopy in conjunction with multivariate statistical techniques. Food Chemistry.

