# Supplementary material for: Rapid traceability of Gastrodia elata Blume origins and analysis of key volatile organic components using FTIR and HS-SPME-GC–MS combined with chemometrics
Source: Food Chem X. 2025 Jul 9;29:102770. doi: 10.1016/j.fochx.2025.102770 (PMC12275067; doi:10.1016/j.fochx.2025.102770)
Supplement: Supplementary material 1 — Supplementary figures and tables for manuscripts. [file mmc1.docx]

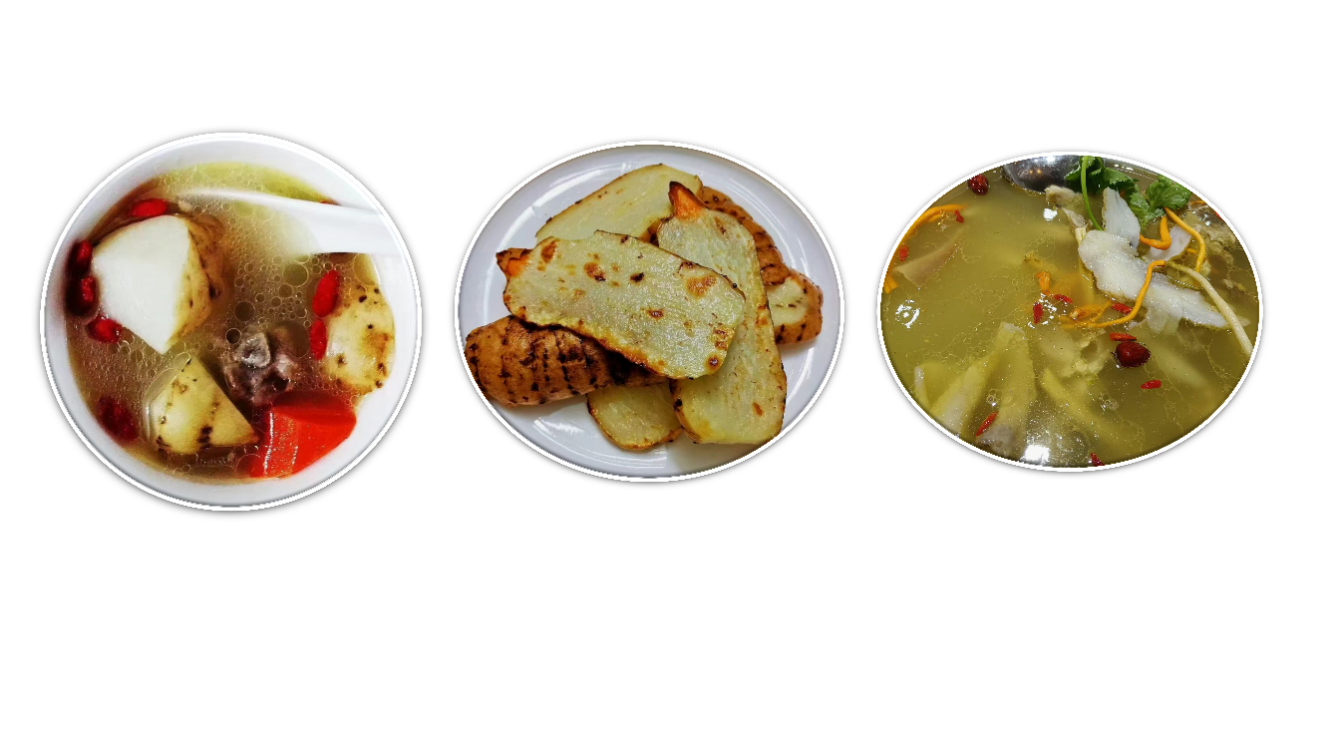


Figure S1. *G. elata* food values


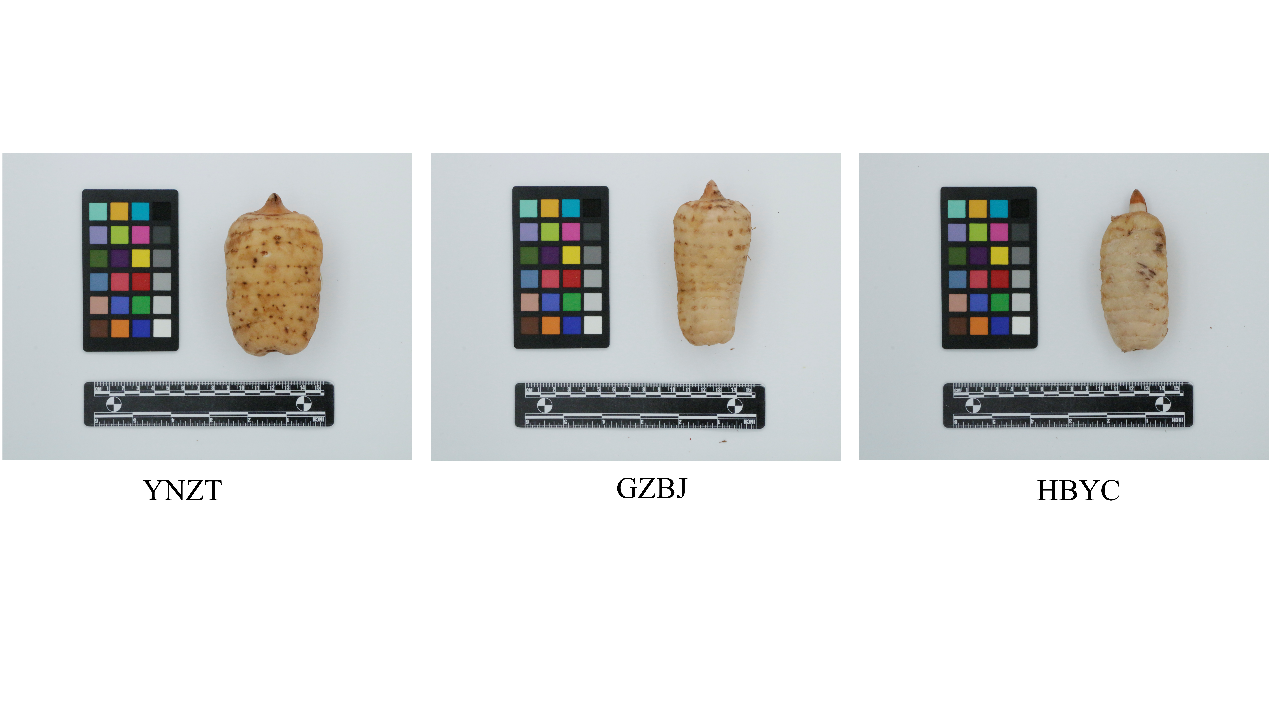
Figure S2. The phenotype of *G. elata* from different origins


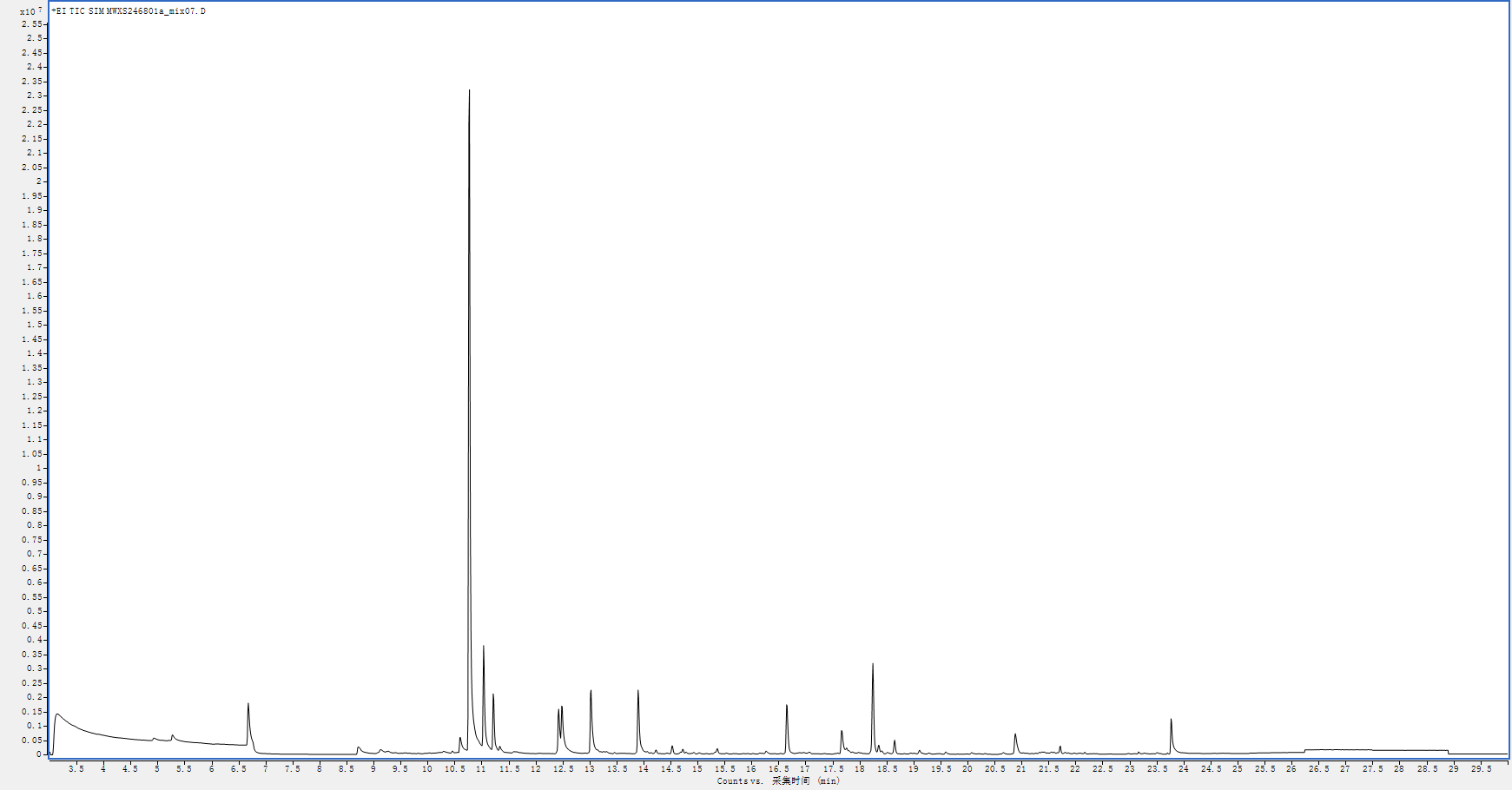


Figure S3. QC sample mass spectrometry detection TIC overlap diagram


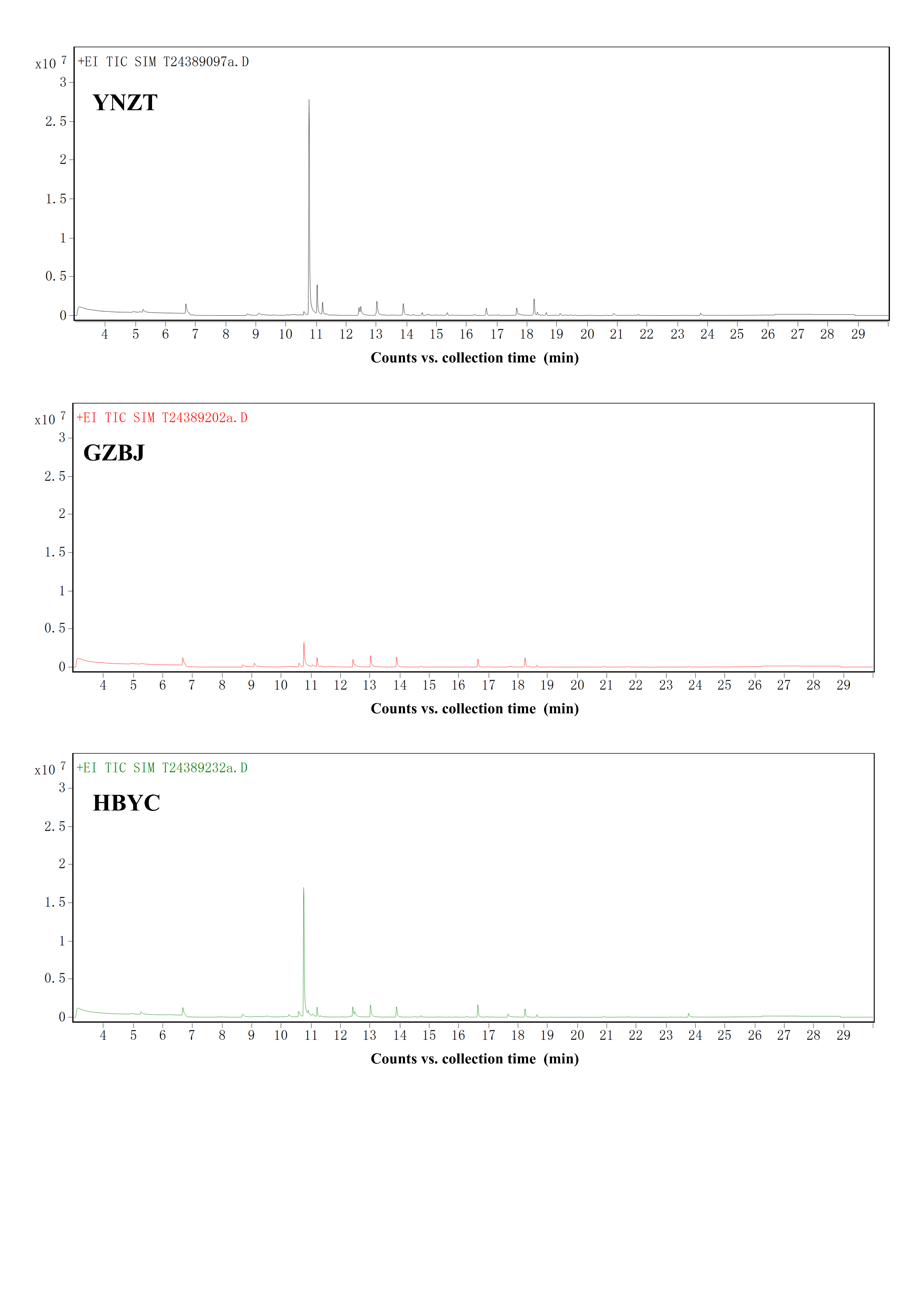


Figure S4. Chromatogram


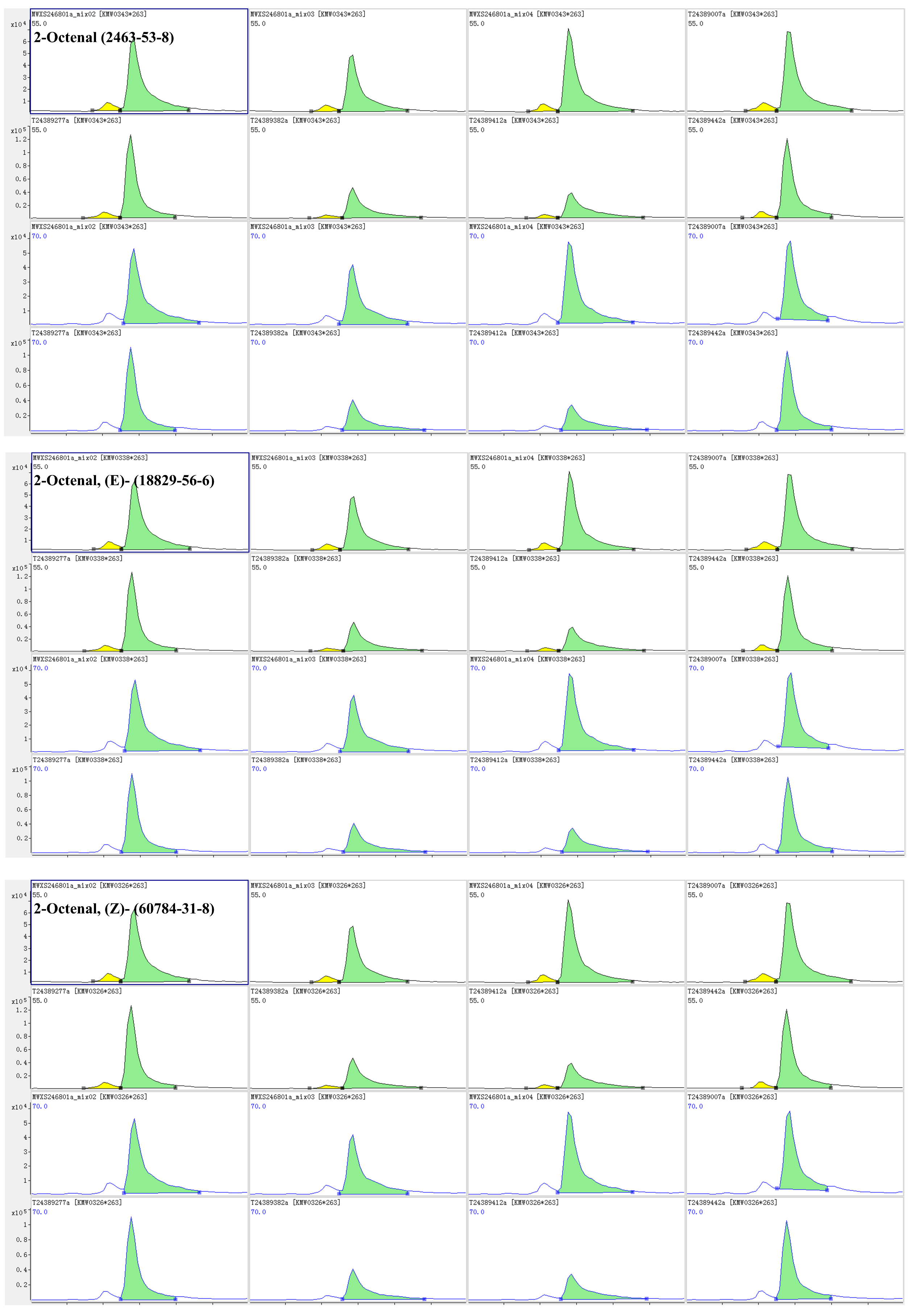


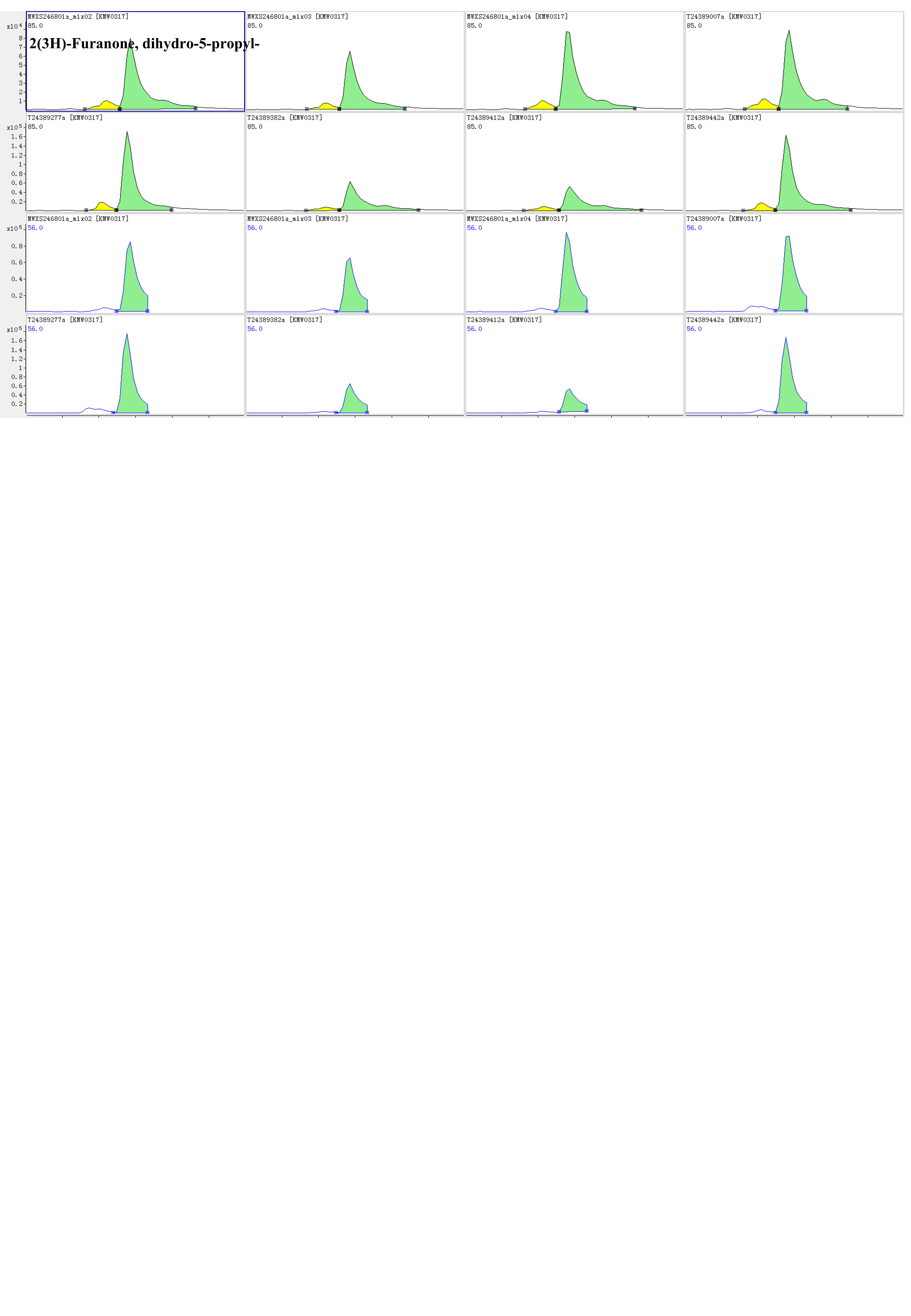


Figure S5. Chromatogram integration diagram


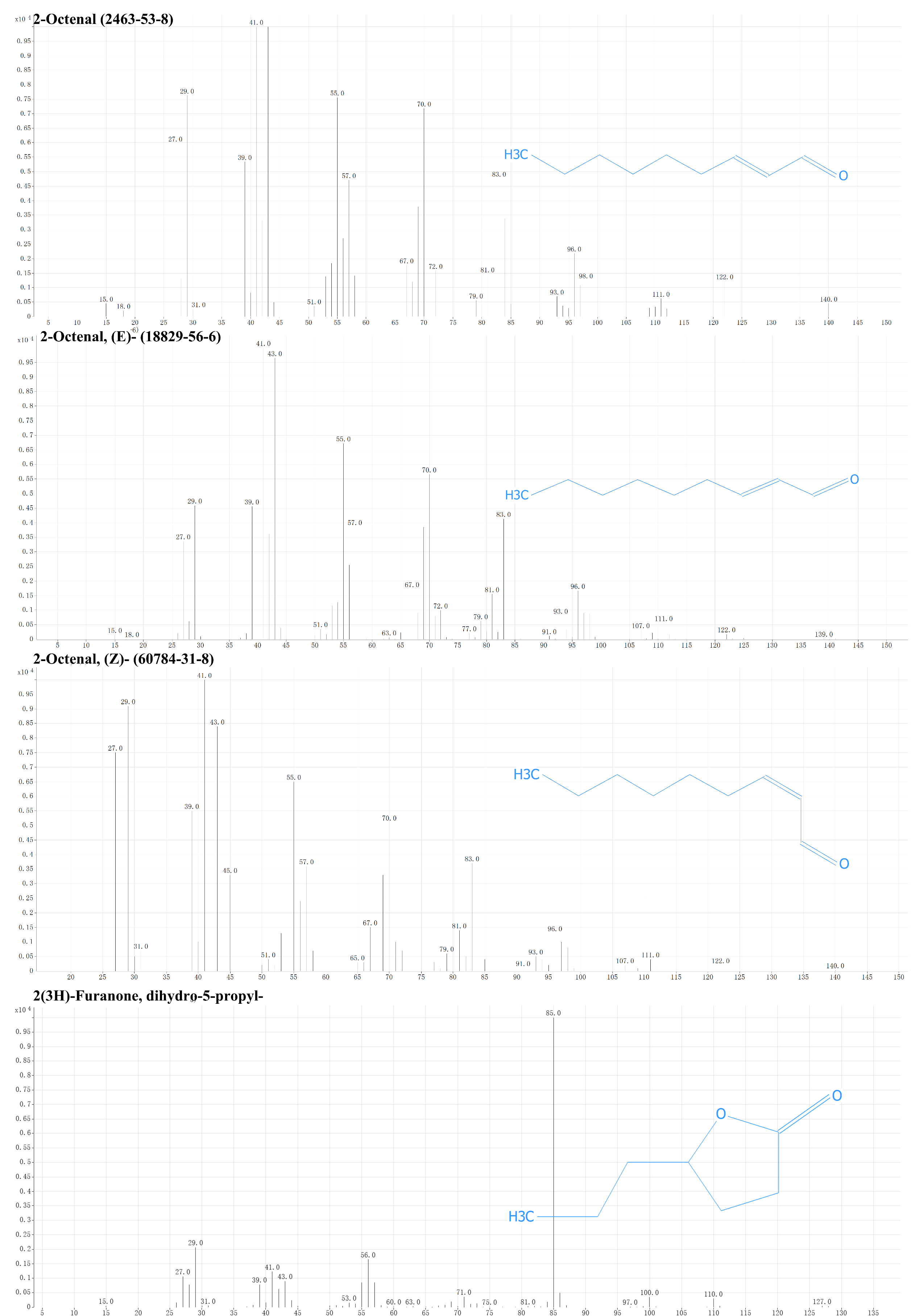


Figure S6. Mass spectrometry diagram


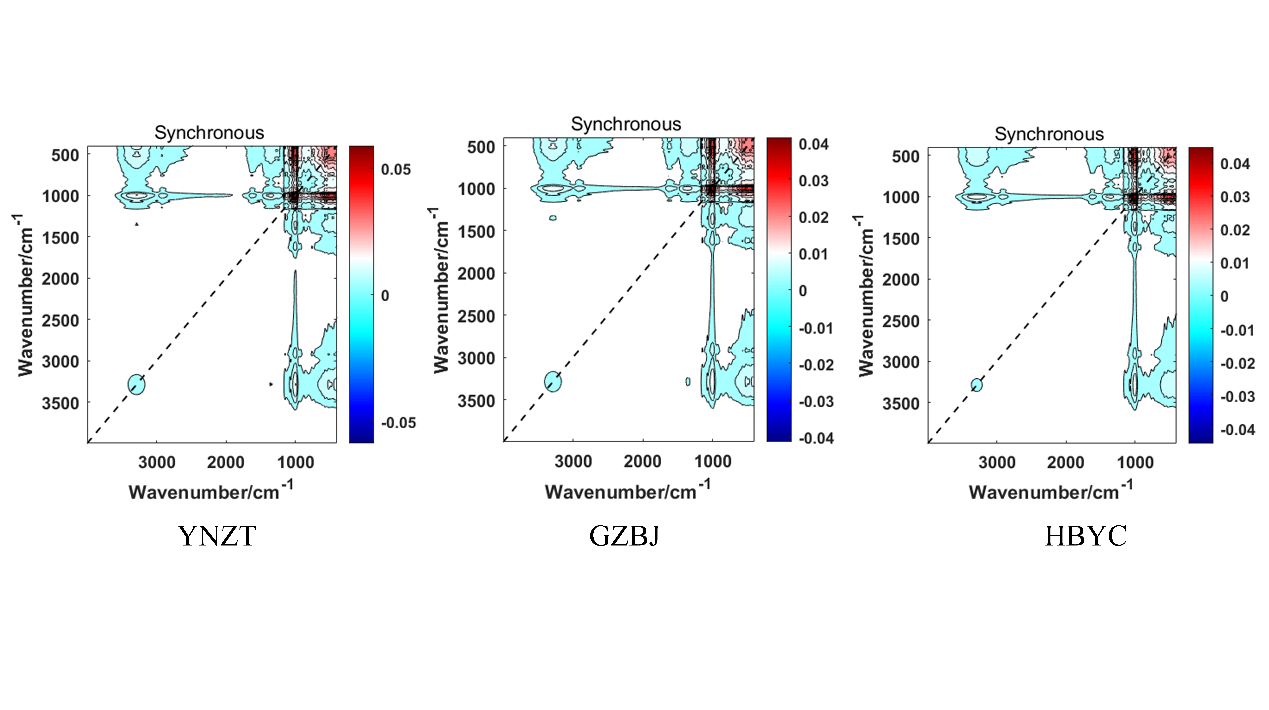
Figure S7. Synchronous 2DCOS images of *G. elata* from different origins. YNZT: Zhaotong, Yunnan Province; GZBJ: Bijie City, Guizhou Province; HBYC: Yichang City, Hubei Province (In the synchronous spectra, the red/white regions (positive correlation) indicate a simultaneous change (stronger or weaker) in the absorption bands, while the blue/blue-green regions (negative correlation) do the opposite).


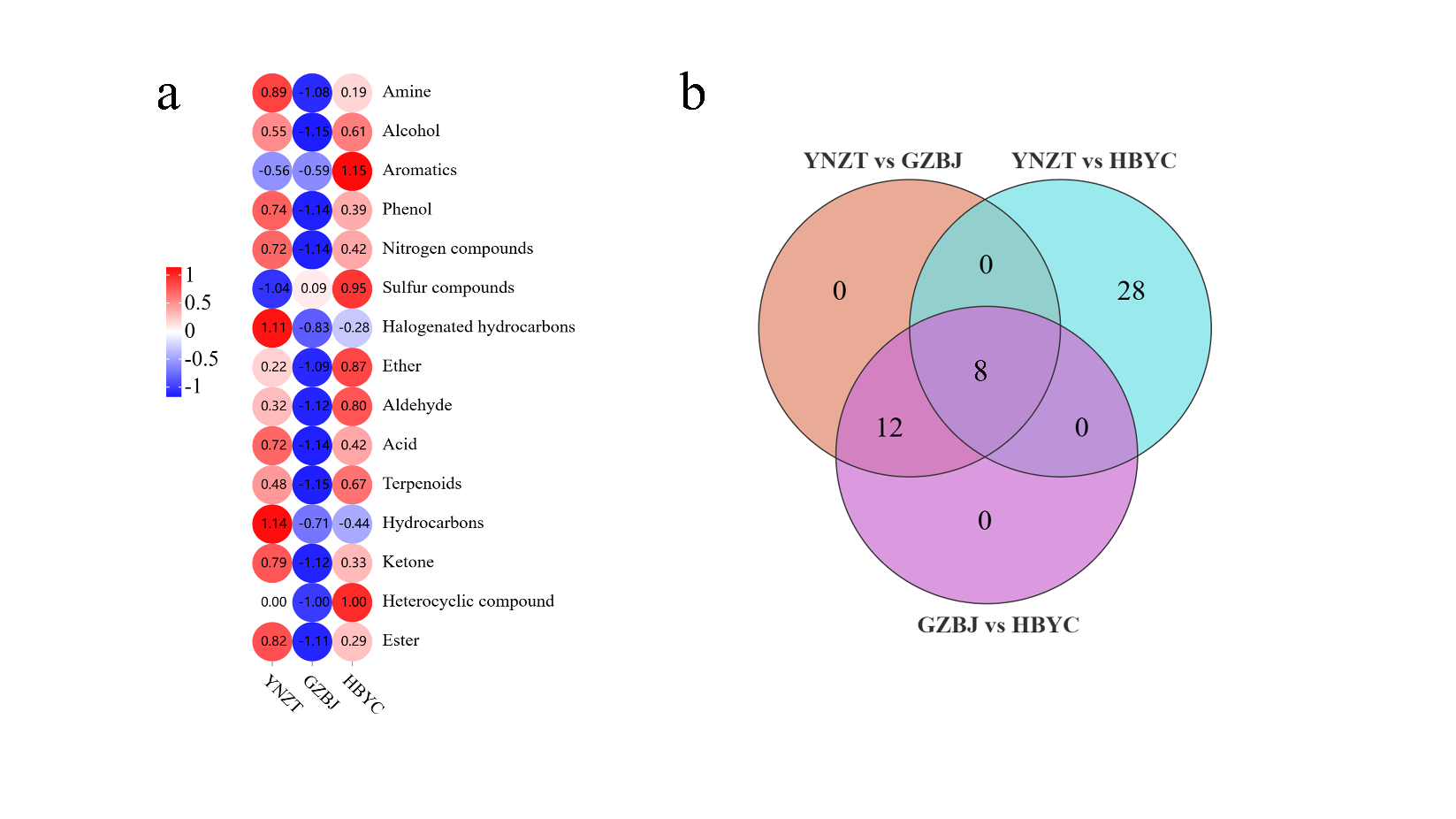


Figure S8. (a) Relative contents of volatile components of *G. elata* from different origins. (b) Venn diagram of differences in major volatile metabolites between the three groups.





Figure S9. Structural formulae of differential VOCs of *G. elata* from YNZT, GZBJ, and HBYC.


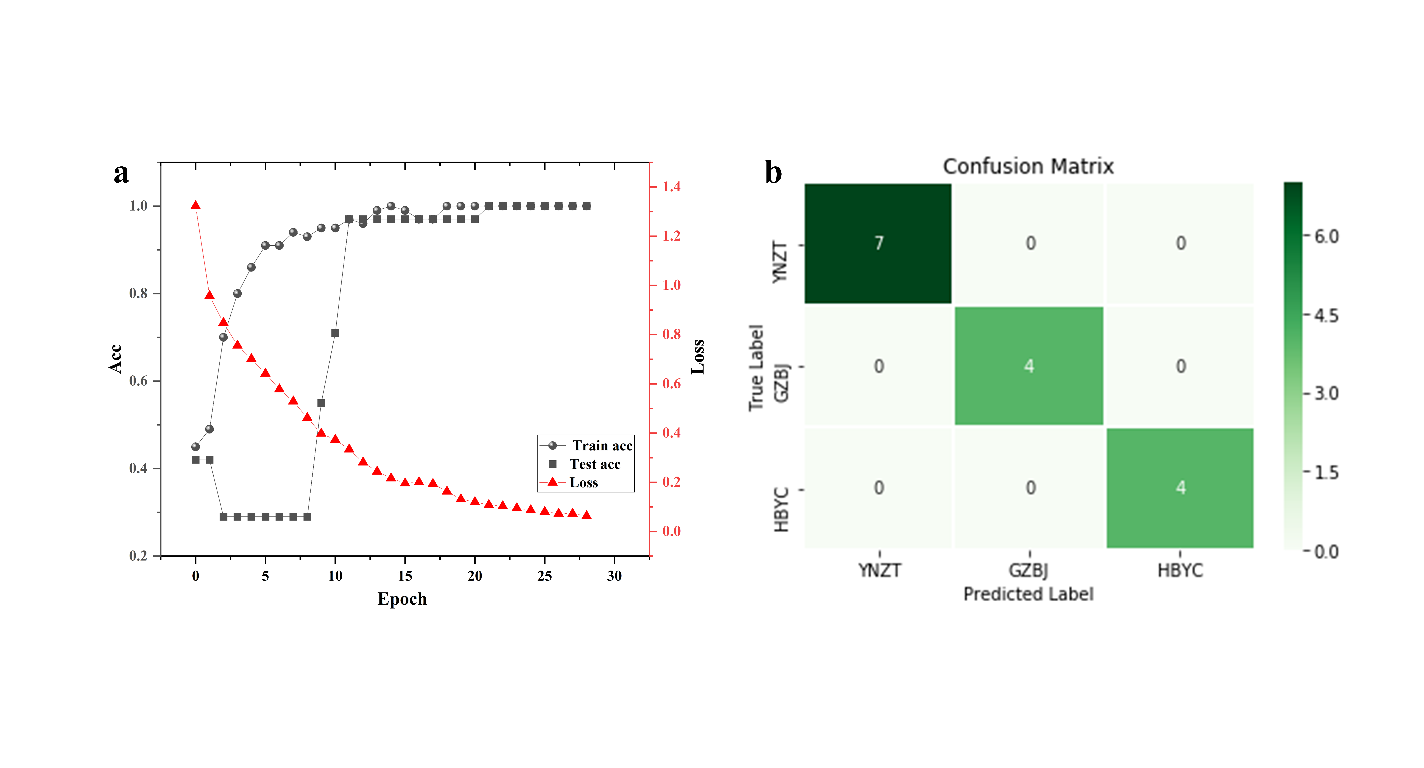


Figure S10. Results of ResNet models constructed based on synchronous 2DCOS images from *G. elata* datasets of different origins. (a) Training set, prediction set accuracy and loss curve function; (b) Confusion proofs for external validation sets.

Table S1. Major volatile components of rOAV > 1 in *G. elata* from different origins.

| **Index** | **Compounds** | **Category** | **Formula** | **Quantitative ion** | **Qualitative ion** | **CAS** | **NIST_RI** | **RT （min）** | **Threshold** | **rOVS** | | | **Aroma Profile** |
| --- | --- | --- | --- | --- | --- | --- | --- | --- | --- | --- | --- | --- | --- |
|  |  |  |  |  |  |  |  |  |  | **YNZT** | **GZBJ** | **HBYC** |  |
| NMW0022 | Benzenemethanol, .alpha.-methyl- | Alcohol | C_8_H_10_O | 79 | 107 | 98-85-1 | 1061.2 | - | 0.479 | 11.20 | 5.81 | 9.16 | fresh, sweet, gardenia, hyacinth |
| KMW0294*237 | 2-Octen-1-ol, (E)- | Alcohol | C_8_H_16_O | 81 | 110 | 18409-17-1 | 1067.6 | - | 0.02 | 21.77 | 12.11 | 17.97 | green, citrus, vegetable, fatty |
| KMW0466 | 3-Cyclohexene-1-methanethiol, .alpha.,.alpha.,4-trimethyl- | Alcohol | C_10_H_18_S | 121 | 136 | 71159-90-5 | 1283 | 14.725 | 2E-08 | 5146765.61 | 4561687.25 | 4358607.60 | sulfury, aromatic, grapefruit, naphthyl, resinous, woody |
| KMW0276 | 1-Octanol | Alcohol | C_8_H_18_O | 56 | 55 | 111-87-5 | 1069.8 | - | 0.022 | 1.95 | 1.29 | 1.33 | intense citrus, rose |
| D94 | Benzenemethanethiol | Alcohol | C_7_H_8_S | 65 | 124 | 100-53-8 | 1092 | 11.214 | 3.5E-06 | 8640.25 | 7391.87 | 7708.37 | sharp, alliaceous, onion, sulfury, garlic, horseradish, minty, coffee |
| KMW0456 | 1-Decanol | Alcohol | C_10_H_22_O | 55 | 70 | 112-30-1 | 1271.5 | - | 0.023 | 2.83 | 2.65 | 2.48 | fatty, waxy, floral, orange, sweet, watery |
| WAMW0925*237 | 2-Octen-1-ol | Alcohol | C_8_H_16_O | 81 | 110 | 22104-78-5 | 1066 | - | 0.05 | 8.71 | 4.84 | 7.19 | green, vegetable |
| WAMW1017*237 | 2-Octen-1-ol, (Z)- | Alcohol | C_8_H_16_O | 81 | 110 | 26001-58-1 | 1067 | - | 0.025 | 17.42 | 9.69 | 14.38 | sweet, floral |
| KMW0404 | Naphthalene | Aromatics | C_10_H_8_ | 128 | 129 | 91-20-3 | 1189.6 | - | 0.05 | 1.62 | 1.39 | 1.41 | pungent, dry, tarry |
| KMW0261*194 | p-Cresol | Phenol | C_7_H_8_O | 108 | 107 | 106-44-5 | 1073.4 | 10.775 | 0.00024 | 160284.46 | 99477.35 | 133265.39 | phenol, narcissus, animalic, mimosa |
| KMW0258 | Phenol, 2-methoxy- | Phenol | C_7_H_8_O_2_ | 109 | 124 | 90-05-1 | 1090.7 | 11.218 | 0.0016 | 173.15 | 152.74 | 159.03 | nutty |
| KMW0271*194 | Phenol, 3-methyl- | Phenol | C_7_H_8_O | 108 | 107 | 108-39-4 | 1075.4 | 10.775 | 0.38 | 101.23 | 62.83 | 84.17 | smoky, petroleum |
| GMW0262 | Octanenitrile | Nitrogen compounds | C_8_H_15_N | 68 | 96 | 124-12-9 | 1081 | 11.215 | 0.00013 | 963.07 | 837.75 | 870.31 | fatty, aldehydic, green |
| KMW0612 | 1,3-Benzodioxole, 4-methoxy-6-(2-propenyl)- | Ether | C_11_H_12_O_3_ | 192 | 91 | 607-91-0 | 1520 | 18.642 | 0.088 | 4.65 | 2.12 | 3.10 | spicy, warm, balsamic, woody |
| WAMW0654 | Benzene, 1,3-dimethoxy- | Ether | C_8_H_10_O_2_ | 109 | 78 | 151-10-0 | 1168 | - | 0.0214 | 2.35 | 2.30 | 2.58 | acid, fruity, nutmeg, neroli |
| KMW0088*005 | 2-Hexenal, (E)- | Aldehyde | C_6_H_10_O | 98 | 42 | 6728-26-3 | 853.2 | 6.683 | 0.0031 | 1876.37 | 1597.67 | 1764.64 | green, grassy |
| QWMW1287 | Non-8-enal | Aldehyde | C_9_H_16_O | 94 | 67 | 39770-04-2 | 1094 | 11.217 | 0.0002 | 1979.18 | 1735.44 | 1802.98 | smoky, plastic |
| KMW0338*263 | 2-Nonenal, (E)- | Aldehyde | C_9_H_16_O | 55 | 70 | 18829-56-6 | 1161.2 | 12.499 | 0.00008 | 6134.41 | 2805.86 | 4835.87 | fatty, green, cucumber, aldehydic, citrus |
| KMW0489 | 1-Cyclohexene-1-carboxaldehyde, 4-(1-methylethenyl)-, (S)- | Aldehyde | C_10_H_14_O | 68 | 79 | 18031-40-8 | 1243 | - | 0.03 | 3.00 | 2.94 | 3.11 | fresh, green, oily, grassy, fatty, minty, cherry |
| KMW0287 | (Z,Z)-3,6-Nonadienal | Aldehyde | C_9_H_14_O | 110 | 67 | 21944-83-2 | 1100 | 11.218 | 0.00005 | 2563.41 | 2261.80 | 2325.06 | fatty, soapy, cucumber |
| KMW0212 | Benzeneacetaldehyde | Aldehyde | C_8_H_8_O | 92 | 91 | 122-78-1 | 1045.6 | - | 0.0063 | 11.46 | 11.35 | 41.46 | floral, honey, rose, cherry |
| KMW0253*230 | 2-octenal | Aldehyde | C_8_H_14_O | 70 | 93 | 2363-89-5 | 1059 | 10.752 | 0.0002 | 103.21 | 75.50 | 81.65 | fatty, green, herbal |
| KMW0095*005 | 2-Hexenal | Aldehyde | C_6_H_10_O | 98 | 42 | 505-57-7 | 851 | 6.683 | 0.017 | 342.16 | 291.34 | 321.79 | sweet, almond, fruity, green, leafy, apple, plum, vegetable |
| KMW0326*263 | 2-Nonenal, (Z)- | Aldehyde | C_9_H_16_O | 55 | 70 | 60784-31-8 | 1148 | 12.499 | 0.0045 | 109.06 | 49.88 | 85.97 | orris, fatty, waxy, cucumber |
| KMW0343*263 | 2-Nonenal | Aldehyde | C_9_H_16_O | 55 | 70 | 2463-53-8 | 1161 | 12.499 | 0.0001 | 4907.53 | 2244.69 | 3868.69 | fatty, green, waxy, cucumber, melon |
| D219*412 | Benzaldehyde, 4-methoxy- | Aldehyde | C_8_H_8_O_2_ | 135 | 136 | 123-11-5 | 1259.8 | 13.898 | 0.0002 | 213.10 | 243.02 | 158.69 | sweet, powdery, mimosa, floral, hawthorn, balsamic |
| KMW0243*230 | 2-Octenal, (E)- | Aldehyde | C_8_H_14_O | 70 | 93 | 2548-87-0 | 1057.4 | - | 0.003 | 6.88 | 5.03 | 5.44 | fresh, cucumber, fatty, green, herbal, banana, waxy, leafy |
| KMW0517*053 | 2-Undecenal, E- | Aldehyde | C_11_H_20_O | 70 | 41 | 53448-07-0 | 1366 | - | 0.00078 | 12.92 | 18.49 | 12.81 | fresh, fruity, citrus, orange, peel |
| XMW0089*454 | 4-Decenal, (E)- | Aldehyde | C_10_H_18_O | 84 | 98 | 65405-70-1 | 1198 | - | 0.025 | 1.87 | 1.14 | 1.12 | fresh, aldehydic, citrus, orange, mandarin, tangerine, green, fatty |
| KMW0432 | Neral | Terpenoids | C_10_H_16_O | 84 | 69 | 106-26-3 | 1240 | - | 1 | 3.56 | 3.48 | 3.77 | sweet, citral, lemon, peel |
| NMW0065*092 | Bicyclo[2.2.1]heptan-2-ol, 1,7,7-trimethyl-, (1S-endo)- | Terpenoids | C_10_H_18_O | 95 | 110 | 464-45-9 | 1170 | - | 0.048 | 28.05 | 27.31 | 29.45 | pine, woody, camphor |
| KMW0439*285 | 2,6-Octadien-1-ol, 3,7-dimethyl-, (Z)- | Terpenoids | C_10_H_18_O | 93 | 69 | 106-25-2 | 1230.5 | - | 0.049 | 4.10 | 3.95 | 4.34 | lemon, fresh |
| KMW0413*279 | (-)-Carvone | Terpenoids | C_10_H_14_O | 54 | 82 | 6485-40-1 | 1249.9 | - | 0.085 | 1.34 | 1.28 | 1.38 | sweet, spearmint, herbal, minty |
| KMW0526 | 2-Buten-1-one, 1-(2,6,6-trimethyl-1,3-cyclohexadien-1-yl)-, (E)- | Terpenoids | C_13_H_18_O | 121 | 69 | 23726-93-4 | 1386 | 16.651 | 0.0015 | 89.61 | 71.64 | 101.36 | apple, rose, honey, tobacco, sweet |
| KMW0639 | 3-Buten-2-one, 4-(2,2,6-trimethyl-7-oxabicyclo[4.1.0]hept-1-yl)- | Terpenoids | C_13_H_20_O_2_ | 123 | 41 | 23267-57-4 | 1473 | - | 0.1 | 1.45 | 1.26 | 1.25 | fruity, sweet, berry, woody, violet, orris, powdery |
| KMW0304 | 1,3,8-p-Menthatriene | Terpenoids | C_10_H_14_ | 119 | 91 | 18368-95-1 | 1119 | - | 0.015 | 3.05 | 2.82 | 2.07 | turpentine, camphor, herbal, woody |
| KMW0389*092 | endo-Borneol | Terpenoids | C_10_H_18_O | 95 | 110 | 507-70-0 | 1170.4 | - | 0.18 | 7.48 | 7.28 | 7.85 | pine, woody, camphor, balsamic |
| KMW0460*285 | Geraniol | Terpenoids | C_10_H_18_O | 93 | 69 | 106-24-1 | 1255.6 | - | 0.0066 | 30.44 | 29.29 | 32.20 | sweet, floral, fruity, rose, waxy, citrus |
| XMW0961*044 | Bornyl acetate | Terpenoids | C_12_H_20_O_2_ | 95 | 93 | 76-49-3 | 1285 | - | 0.075 | 2.63 | 2.43 | 2.44 | woody, pine, herbal, cedary, spice |
| KMW0444*279 | Carvone | Terpenoids | C_10_H_14_O | 54 | 82 | 99-49-0 | 1242 | - | 0.067 | 1.70 | 1.62 | 1.75 | minty, licorice |
| KMW0448*279 | D-Carvone | Terpenoids | C_10_H_14_O | 54 | 82 | 2244-16-8 | 1246 | - | 0.01 | 11.41 | 10.88 | 11.72 | spice, minty, bread, caraway |
| KMW0459*283 | 2,6-Octadienal, 3,7-dimethyl-, (E)- | Terpenoids | C_10_H_16_O | 69 | 84 | 141-27-5 | 1270 | - | 0.028 | 2.95 | 2.23 | 2.51 | citrus, lemon |
| KMW0603*061 | Germacrene D | Terpenoids | C_15_H_24_ | 161 | 105 | 23986-74-5 | 1481 | 18.249 | 0.0012 | 110.25 | 69.83 | 88.31 | woody, spice |
| XMW0462 | 2-Cyclopenten-1-one, 3-methyl-2-(2-pentenyl)-, (Z)- | Ketone | C_11_H_16_O | 164 | 79 | 488-10-8 | 1395 | 16.654 | 0.00026 | 4023.91 | 2958.49 | 3424.28 | woody, herbal, floral, spicy, jasmin, celery |
| KMW0383 | 2(5H)-Furanone, 5-ethyl-3-hydroxy-4-methyl- | Ketone | C_7_H_10_O_3_ | 97 | 57 | 698-10-2 | 1195 | 13.023 | 0.000002 | 313012.07 | 295905.66 | 311684.69 | sweet, fruity, caramel, maple, fenugreek, brown, sugar, nutty, chicory, praline, butterscotch |
| QWMW0607 | 3-Nonanone | Ketone | C_9_H_18_O | 85 | 113 | 925-78-0 | 1090 | - | 0.017 | 14.24 | 14.29 | 15.48 | caramel, spicy, sweet |
| WMW0176 | 2H-Pyran-2-one, tetrahydro-6-methyl- | Ketone | C_6_H_10_O2 | 70 | 42 | 823-22-3 | 1095.1 | - | 0.02683 | 7.15 | 6.64 | 7.61 | creamy, fruity, coconut |
| w21 | 2H-Pyran-2-one, 6-pentyl- | Ketone | C_10_H_14_O_2_ | 95 | 82 | 27593-23-3 | 1453 | - | 0.15 | 4.15 | 1.00 | 2.27 | tonka, lactonic, coconut, creamy, fatty, waxy |
| KMW0230*229 | 3-Octen-2-one | Ketone | C_8_H_14_O | 55 | 111 | 1669-44-9 | 1040 | 10.256 | 0.00003 | 3181.47 | 3447.38 | 4223.13 | earthy, spicy, herbal, sweet, mushroom, hay, blueberry |
| KMW0539 | 2(5H)-Furanone, 5-ethyl- | Ketone | C_6_H_8_O_2_ | 83 | 112 | 2407-43-4 | 966 | - | 0.0097 | 9.94 | 9.96 | 11.64 | spice |
| WAMW1925 | 2-Decanone | Ketone | C_10_H_20_O | 43 | 156 | 693-54-9 | 1193 | - | 0.06 | 2.80 | 1.61 | 1.63 | orange, floral, fatty, peach |
| NMW0281 | 3-Butylisobenzofuran-1(3H)-one | Ketone | C_12_H_14_O_2_ | 133 | 105 | 6066-49-5 | 1656 | - | 0.01 | 3.04 | 2.13 | 2.60 | herbal, phenol, celery |
| XMW0186*139 | 3,5-Octadien-2-one, (E,E)- | Ketone | C_8_H_12_O | 95 | 43 | 30086-02-3 | 1073 | 10.774 | 0.0005 | 1301.56 | 1141.91 | 1179.22 | fruity, green, grassy |
| KMW0563*085 | 5,9-Undecadien-2-one, 6,10-dimethyl-, (E)- | Ketone | C_13_H_22_O | 69 | 151 | 3796-70-1 | 1453 | - | 0.01 | 2.71 | 3.57 | 2.68 | fresh, green, fruity, waxy, rose, woody, magnolia, tropical |
| GMW0285*227 | 3,4-Dimethyl-1,2-cyclopentadione | Ketone | C_7_H_10_O_2_ | 126 | 111 | 13494-06-9 | 1109 | - | 0.017 | 2.09 | 1.35 | 1.54 | sweet, maple, caramel, sugar, fenugreek, licorice |
| KMW0351 | Pyrazine, 2,3-diethyl-5-methyl- | Heterocyclic compound | C_9_H_14_N_2_ | 150 | 135 | 18138-04-0 | 1158 | 12.401 | 0.000031 | 299.41 | 51.50 | 900.97 | musty, nut skin, earthy, roasted, hazelnut, toasted, potato, dusty, foliage, vegetable |
| QWMW0245 | 1,3-Dithiolo[4,5-b]furan, tetrahydro-3a-methyl- | Heterocyclic compound | C_6_H_10_OS_2_ | 71 | 84 | 67411-25-0 | 1245 | 13.877 | 0.006 | 16.65 | 9.01 | 10.31 | boiled, milky, chicken, cooked beef, rubbery, sulfury, thiamin |
| KMW0188 | 2-Methyl-1,3-dithiacyclopentane | Heterocyclic compound | C_4_H_8_S_2_ | 120 | 105 | 5616-51-3 | 1026 |  | 0.02 | 2.07 | 1.46 | 2.05 | sulfury, alliaceous, smoky, savory, vegetable |
| KMW0268*022 | Pyrazine, 2-ethyl-3,5-dimethyl- | Heterocyclic compound | C_8_H_12_N_2_ | 135 | 136 | 13925-07-0 | 1084 | 11.07 | 0.00004 | 130.76 | 3.77 | 8.66 | burnt, almond, roasted, nutty, coffee |
| XMW0007 | Dibutyl phthalate | Ester | C_16_H_22_O_4_ | 149 | 150 | 84-74-2 | 1972.6 |  | 0.26 | 1.67 | 0.72 | 1.28 | faint |
| KMW0317 | 2(3H)-Furanone, dihydro-5-propyl- | Ester | C_7_H_12_O_2_ | 85 | 56 | 105-21-5 | 1156 | 12.498 | 0.4 | 1.71 | 0.83 | 1.38 | sweet, coconut, nutty, caramel, tonka, hay |
| WAMW1792 | 2-Pentanol, acetate | Ester | C_7_H_14_O_2_ | 70 | 87 | 626-38-0 | 849 | - | 0.015 | 19.84 | 16.75 | 18.11 | herbal, weedy, musty, green, vegetable, nut skin, beany, ketonic, animalic |
| D147*110 | Butanoic acid, 3-methyl-, phenylmethyl ester | Ester | C_12_H_16_O_2_ | 91 | 108 | 103-38-8 | 1396 | - | 0.01 | 25.15 | 19.12 | 21.62 | sweet, fruity, apple, pineapple, herbal |
| XMW1240 | Hexanoic acid, 2-methylbutyl ester | Ester | C_11_H_22_O_2_ | 70 | 99 | 2601-13-0 | 1247 | - | 0.032 | 3.87 | 3.75 | 4.00 | ethereal |
| XMW0464 | Benzoic acid, 2-(methylamino)-, methyl ester | Ester | C_9_H_11_NO_2_ | 165 | 105 | 85-91-6 | 1408 | - | 0.0203 | 8.45 | 7.20 | 7.34 | fruity, musty, sweet, neroli, powdery, phenol, wine |
| KMW0421 | Methyl salicylate | Ester | C_8_H_8_O_3_ | 64 | 92 | 119-36-8 | 1192 | - | 0.04 | 1.24 | 1.20 | 1.22 | caramel, pepperminty |
| KMW0303*301 | 2-Propenoic acid, 3-phenyl-, methyl ester | Ester | C_10_H_10_O_2_ | 131 | 103 | 103-26-4 | 1389.3 | - | 0.011 | 7.70 | 2.20 | 2.98 | sweet, balsamic, strawberry, cherry, cinnamon |
| XMW1209 | Propanoic acid, 2-methyl-, 2-methylbutyl ester | Ester | C_9_H_18_O_2_ | 71 | 43 | 2445-69-4 | 1016 | - | 0.014 | 3.07 | 2.92 | 3.05 | fruity, ethereal, tropical, banana |
| KMW0267 | Benzoic acid, methyl ester | Ester | C_8_H_8_O_2_ | 105 | 77 | 93-58-3 | 1097.7 | - | 0.00052 | 32.69 | 31.90 | 26.39 | phenol, wintergreen, almond, floral, canga |
| XMW1401*042 | n-Valeric acid cis-3-hexenyl ester | Ester | C_11_H_20_O_2_ | 82 | 67 | 35852-46-1 | 1237 | - | 0.06 | 5.91 | 5.77 | 6.20 | green, fruity, apple, pear, kiwi, unripe, banana, tropical |
| XMW1403*042 | cis-3-Hexenyl isovalerate | Ester | C_11_H_20_O_2_ | 82 | 67 | 35154-45-1 | 1238 | - | 0.02 | 17.74 | 17.30 | 18.61 | fresh, green, apple, fruity, tropical, pineapple |
| XMW0981*126 | Butanoic acid, 3-methyl-, 2-phenylethyl ester | Ester | C_13_H_18_O_2_ | 104 | 57 | 140-26-1 | 1491 | 18.24 | 0.00001 | 3240.46 | 1889.06 | 2534.51 | floral, fruity, sweet, rose, peach, apricot |

Table S2. OPLS-DA modeling is based on different preprocessing methods and their combinations.

| Pretreatment | LVs | R^2^X | R^2^Y | Q^2^ | RMSE_E_ | RMSE_CV_ | RMSE_P_ | Train ACC (%) | Test ACC (%) |
| --- | --- | --- | --- | --- | --- | --- | --- | --- | --- |
| RAW | 6 | 0.996 | 0.448 | 0.404 | 0.309 | 0.342 | 0.283 | 81.67 | 80.43 |
| SG | 6 | 0.996 | 0.452 | 0.404 | 0.309 | 0.342 | 0.281 | 80.00 | 80.43 |
| MSC | 2 | 0.983 | 0.885 | 0.690 | 0.155 | 0.249 | 0.284 | 99.17 | 91.30 |
| SNV | 2 | 0.984 | 0.884 | 0.687 | 0.156 | 0.249 | 0.278 | 99.17 | 91.30 |
| **1st Der** | **2** | **0.711** | **0.939** | **0.760** | **0.121** | **0.210** | **0.241** | **100** | **91.30** |
| 2nd Der | 2 | 0.467 | 0.963 | 0.817 | 0.098 | 0.202 | 0.183 | 100 | 95.65 |

Table S3. The PLSR model of 2-Nonenal (a) and 2(3H)-Furanone, dihydro-5-propyl- based on different pretreatment methods.

| Volatile components | Pretreatment | LVs | Calibration | | | Prediction | | |
| --- | --- | --- | --- | --- | --- | --- | --- | --- |
|  |  |  | R^2^_C_ | RMSE_C_ | RPD_C_ | R^2^_P_ | RMSE_P_ | RPD_P_ |
| 2-Nonenal | Raw | 1 | 0.0513 | 0.1312 | 1.0267 | 0.1235 | 0.1293 | 1.0719 |
|  | SG | 1 | 0.0513 | 0.1312 | 1.0267 | 0.1235 | 0.1293 | 1.0719 |
|  | MSC | 3 | 0.5527 | 0.0901 | 1.4953 | 0.7013 | 0.0740 | 1.8729 |
|  | SNV | 3 | 0.5542 | 0.0900 | 1.4977 | 0.7041 | 0.0736 | 1.8831 |
|  | **1stDer** | **3** | **0.9304** | **0.0356** | **3.7897** | **0.8368** | **0.0565** | **2.4530** |
|  | 2ndDer | 5 | 0.9880 | 0.0248 | 5.4352 | 0.8258 | 0.0572 | 2.4230 |
| 2(3H)-Furanone, dihydro-5-propyl- | Raw | 1 | 0.0721 | 0.1874 | 1.0381 | 0.0725 | 0.1756 | 1.0636 |
|  | SG | 1 | 0.0721 | 0.1874 | 1.0381 | 0.0725 | 0.1756 | 1.0636 |
|  | MSC | 5 | 0.7757 | 0.0921 | 2.1114 | 0.8580 | 0.0881 | 2.1200 |
|  | SNV | 5 | 0.7734 | 0.0926 | 2.1007 | 0.8610 | 0.0877 | 2.1296 |
|  | **1stDer** | **3** | **0.9123** | **0.0576** | **3.3772** | **0.8035** | **0.0823** | **2.2694** |
|  | 2ndDer | 5 | 0.9835 | 0.0250 | 7.7965 | 0.7537 | 0.0905 | 2.0637 |

Table S4. The PLSR model of 2-Nonenal and 2(3H)-Furanone, dihydro-5-propyl- based on VIP feature extraction.

| Volatile components | Datasets | LVs | Calibration | | | Prediction | | |
| --- | --- | --- | --- | --- | --- | --- | --- | --- |
|  |  |  | R^2^_C_ | RMSE_C_ | RPD_C_ | R^2^_P_ | RMSE_P_ | RPD_P_ |
| 2-Nonenal | Raw-VIP | 1 | 0.0630 | 0.1304 | 1.0331 | 0.1294 | 0.1286 | 1.0777 |
|  | **1stDer-VIP** | **4** | **0.9779** | **0.0200** | **6.7261** | **0.8630** | **0.0533** | **2.6003** |
| 2(3H)-Furanone, dihydro-5-propyl- | Raw-VIP | 1 | 0.0845 | 0.1861 | 1.0451 | 0.816 | 0.1749 | 1.0679 |
|  | **1stDer-VIP** | **3** | **0.9218** | **0.0544** | **3.5768** | **0.8161** | **0.0782** | **2.3883** |
